# The MsNAC73–MsMPK3 Complex Modulates Salt Tolerance and Shoot Branching of Alfalfa via Activating *MsPG2* and *MsPAE12* Expressions

**DOI:** 10.1111/pbi.70323

**Published:** 2025-08-20

**Authors:** Xiangkai You, Nana Fan, Yuehua Zhang, Linjie Sun, Liantai Su, Wuwu Wen, Aimin Lv, Xinyu Dai, Li Gao, Fengling Shi, Peng Zhou, Zhaoming Wang, Yuan An

**Affiliations:** ^1^ School of Agriculture and Biology Shanghai Jiao Tong University Shanghai P. R. China; ^2^ Department of Biomedical Sciences City University of Hong Kong Hong Kong SAR P. R. China; ^3^ College of Advanced Agricultural Sciences Yulin University Yulin Shaanxi P. R. China; ^4^ Technology R&D Center, M‐Grass Ecology and Environment (Group) Co. Ltd Hohhot Inner Mongolia P. R. China; ^5^ College of Grassland and Resources and Environment Inner Mongolia Agricultural University Hohhot Inner Mongolia P. R. China; ^6^ College of Grassland Agriculture Northwest A&F University Yangling Shaanxi P. R. China; ^7^ National Key Laboratory for Development and Utilization of Forest Food Resources Zhejiang A&F University Zhejiang Hangzhou P. R. China; ^8^ Key Laboratory of Urban Agriculture, Ministry of Agriculture and Rural Affairs Shanghai P. R. China

**Keywords:** alfalfa, NAC transcription factor, pectin acetylesterase, phosphorylation, polygalacturonase, salt stress

## Abstract

Balancing plant growth and survival is an important strategy for plants to adapt to different environments. However, the molecular mechanisms of the balance strategy are poorly understood. Our previous study demonstrated that MsNAC73 interacts with the promoter of *MsPAE12*, which positively regulates alfalfa shoot branching. In the present study, MsNAC73 was further found to interact with the promoter of *MsPG2* and negatively regulate *MsPG2* expression through MODMS database analysis and experimental verification (Y1H, EMSA and Dual‐LUC assays). The transgenic alfalfa plants with overexpression or knockdown of *MsNAC73* and *MsPG2* were obtained, and their responses to abiotic stress were analysed. Overexpressing *MsNAC73* negatively and overexpressing *MsPG2* positively affected the salt tolerance of alfalfa. MsPG2 increased salt tolerance via hydrolysing pectins, increasing cell wall extensibility and reducing Na^+^/K^+^ ratio, stomatal aperture and vessel diameter. Co‐IP, Y2H, split‐LUC and BiFC assays demonstrated that MsMPK3 interacted with MsNAC73 and phosphorylated MsNAC73 at the Thr‐123 site. Furthermore, low abundances of MsMPK3 and MsNAC73 under normal conditions diminished the MsNAC73 phosphorylation, thereby promoting *MsPAE12* expression and increasing alfalfa shoot branching. Under salt stress, however, MsNAC73 and MsMPK3 were upregulated at transcript and protein levels. The increased phosphorylation of MsNAC73 (MsNAC73^T123D^) promoted *MsPG2* expression. Additionally, overexpression of *MsNAC73* ^*T123D*^ and *MsNAC73* ^*T123A*^ (dephosphorylation of MsNAC73) in alfalfa hairy roots increased root elongation under salt conditions and lateral root amounts under normal conditions respectively. In brief, these results revealed that the MsNAC73‐MsMPK3‐*MsPG2/MsPAE12* module plays a key role in the trade‐off between shoot branching and plant survival in response to different environments.

## 1 Introduction

Salt stress is an important environmental factor that severely impairs plant growth and agricultural productivity. This impairment primarily stems from reduced available soil water and excessive Na^+^ and Cl^−^ accumulation in plants (Zhu 2002). In recent years, more evidence has demonstrated that the cell wall plays a crucial role in plant resistance to salt stress (Colin et al. 2023; Endler et al. 2015; Feng et al. 2018; Zhou et al. 2024). The cell wall predominantly comprises celluloses, hemicelluloses and pectins, in which the pectins possess negative charge characteristics and exhibit reversible cation‐binding capacity, which strongly affects Na^+^ accumulation in the cell wall (Anderson and Kieber 2020; Liu et al. 2021; Shomer et al. 2003). Polygalacturonases (PGs), members of the glycosyl hydrolase 28 family, catalyse pectin cleavage in its homogalacturonan (HG) backbones, thereby hydrolysing pectins, changing the structure and function of cell walls and affecting plant responses to various abiotic stresses (Liu et al. 2014; Zhang, Hou, et al. 2021; Zhang, Guo, et al. 2021). Upregulated expressions of *AtPGL1* in Arabidopsis ( *Arabidopsis thaliana* ) and *OsPG2* in rice ( *Oryza sativa* ) reduce the abundance and molecular mass of pectins in the cell wall and increase the *O. sativa* resistance to blast fungus (Ohara et al. 2021; Shen et al. 2025). However, the mechanisms underlying pectins affecting plant salt stress responses have seldom been reported.

NAC (NAM, ATAF1/2 and CUC2) transcription factors (TFs) are one of the most prominent plant‐specific transcription factor families and participate in several processes of plant growth, development and abiotic stress response (Olsen et al. 2005; Xiong et al. 2025). For example, PbNAC71 in pear (*Pyrus* spp.) interacts with the E3 ubiquitin ligase PbRING finger protein 217 (PbRNF217), which facilitates the ubiquitin‐mediated degradation of PbNAC71 via the 26S proteasome pathway, thus regulating the development of xylem and vessels (Cong et al. 2024). Hormones are one of the main elements in plant regulation. An important mechanism of NACs regulating plant development is through the hormone pathway. OsNAC2 directly binds to the promoters of auxin (IAA) related genes *GH3.6*, *GH3.8* and *OsARF25*, and a cytokinin (CTK) oxidase gene *OsCKX4*, thereby participating in the modulation of the root development progress of *O. sativa* via IAA and CTK pathways (Mao et al. 2020). NAC TF SOMBRERO (SMB) mediates root halotropism and directs root growth away from high salinity by establishing asymmetric auxin distribution within the lateral root cap (Zheng et al. 2024). NACs are a large group of TFs participating in plant response to salinity stress. Overexpression of *ShNAC1* in tomato ( *Solanum habrochaites* ), *PeNAC045* in *Populus euphratica*, *OsNAC2* in *O. sativa* , *AtNAP* in *A. thaliana* and a transmembrane domain‐containing NAC TF *TaSIP1* in wheat ( *Triticum aestivum* ) inhibits plant tolerance to salinity stress (Liu et al. 2018; Lu et al. 2018; Mao et al. 2018; Seok et al. 2017; Wang et al. 2022). However, studies regarding NACs related to salt stress response in alfalfa are largely unknown.

Mitogen‐activated protein kinases (MPK or MAPK) represent highly conserved and functionally significant signalling modules in eukaryotes that play critical roles in plant development, immune responses and stress responses (Meng and Zhang 2013; Xu and Zhang 2015; Zhang et al. 2018). The canonical MAPK module comprises three types of kinases: an MPK (MAPK), a MAPK kinase (MAPKK) and a MAPKK kinase (MAPKKK) (Chardin et al. 2017). The MPKs can affect their interacted proteins in independent or cascade methods (Xiang et al. 2021; Zhang and Zhang 2022). OsMAPK3‐mediated phosphorylation of OsbHLH002 positively regulates the expression of *OsTPP1*, thereby enhancing trehalose accumulation and chilling resistance in *O. sativa* (Zhang et al. 2017). MPK3 and MPK6 negatively regulate the stabilities of *A. thaliana* response regulator proteins ARR1, ARR10 and ARR12 to enhance the salt tolerance of plants (Yan et al. 2021). ZmMPK5 phosphorylates Thr‐26 in ZmNAC49, activating *ZmSOD3* expression and enhancing oxidative stress resistance in maize ( *Zea mays* ) (Xiang et al. 2021). In many cases, however, MPKs regulate proteins in the cascade method. The MAPKKK28‐MKK1‐MPK1 cascade participates in the regulation of the abscisic acid (ABA) signalling pathway and enhances *O. sativa* tolerance to oxidative and osmotic stresses, while the OsMAPKKK63‐OsMKK1‐OsMPK4 cascade regulates *O. sativa* tolerance to salt stress by upregulating *OsDREB2A* and *OsDREB2B* expression (Na et al. 2019; Ren et al. 2024; Wang et al. 2014). Recently, an abiotic stress RNA‐seq analysis revealed that the alfalfa MAPK gene family responds to salt, cold and drought stresses, but no more experimental evidence is provided (Liu et al. 2024).

Alfalfa ( *Medicago sativa* ) is one of the most important forage legumes cultivated worldwide (Zhang, Xue, et al. 2023). Our previous studies identified a NAC‐domain transcription factor, designated MsNAC73, from alfalfa. MsNAC73 is demonstrated to be a crucial transcriptional repressor of pectin acetylesterase 12 (*MsPAE12*), which affects alfalfa shoot branching by reducing pectin acetylation to decrease IAA biosynthesis in the apex (Fan et al. 2024). Knockdown of *MsNAC73* (*MsNAC73*‐RNAi) increased alfalfa shoot branching; at the same time, an unexpected result was observed from abiotic stress assays. The salt tolerance of *MsNAC73*‐RNAi lines significantly and strikingly increased. Thus, we speculated that MsNAC73 might regulate other key target genes participating in salt response, and the target genes might be related to pectins. Subsequently, through alfalfa MODMS database analysis (https://modms.lzu.edu.cn) and yeast one‐hybrid (Y1H) screening, a polygalacturonase gene (*MsPG2*), the key pectin hydrolases in plants, was identified as the other downstream target gene of MsNAC73. Notably, the expressions of both *MsNAC73* and *MsPG2* were significantly induced by NaCl stress, suggesting that they might be involved in salt stress response. In the present study, the gain‐ and loss‐of‐function techniques were used to investigate the roles of MsNAC73 and MsPG2 in mediating salt tolerance of alfalfa, and it was found that MsNAC73 inhibited *MsPG2* expression by directly binding to its promoter. Overexpression of *MsPG2* positively affected alfalfa tolerance to salt stress, and a consistent salt‐induced phenotype was observed in *MsNAC73*‐RNAi lines. Furthermore, a protein–protein interaction between MsNAC73 and MsMPK3 was identified. Under normal conditions, the interaction of MsNAC73 and MsMPK3 was weakened, which upregulated *MsPAE12* expression and increased alfalfa shoot branching, while the interaction was strengthened under salt stress, leading to upregulated *MsPG2* expression and increased alfalfa tolerance to salt stress.

## 2 Results

### 2.1 MsNAC73 Negatively Affects Shoot Branching and Salt Stress Tolerance of Alfalfa

**FIGURE 1:**
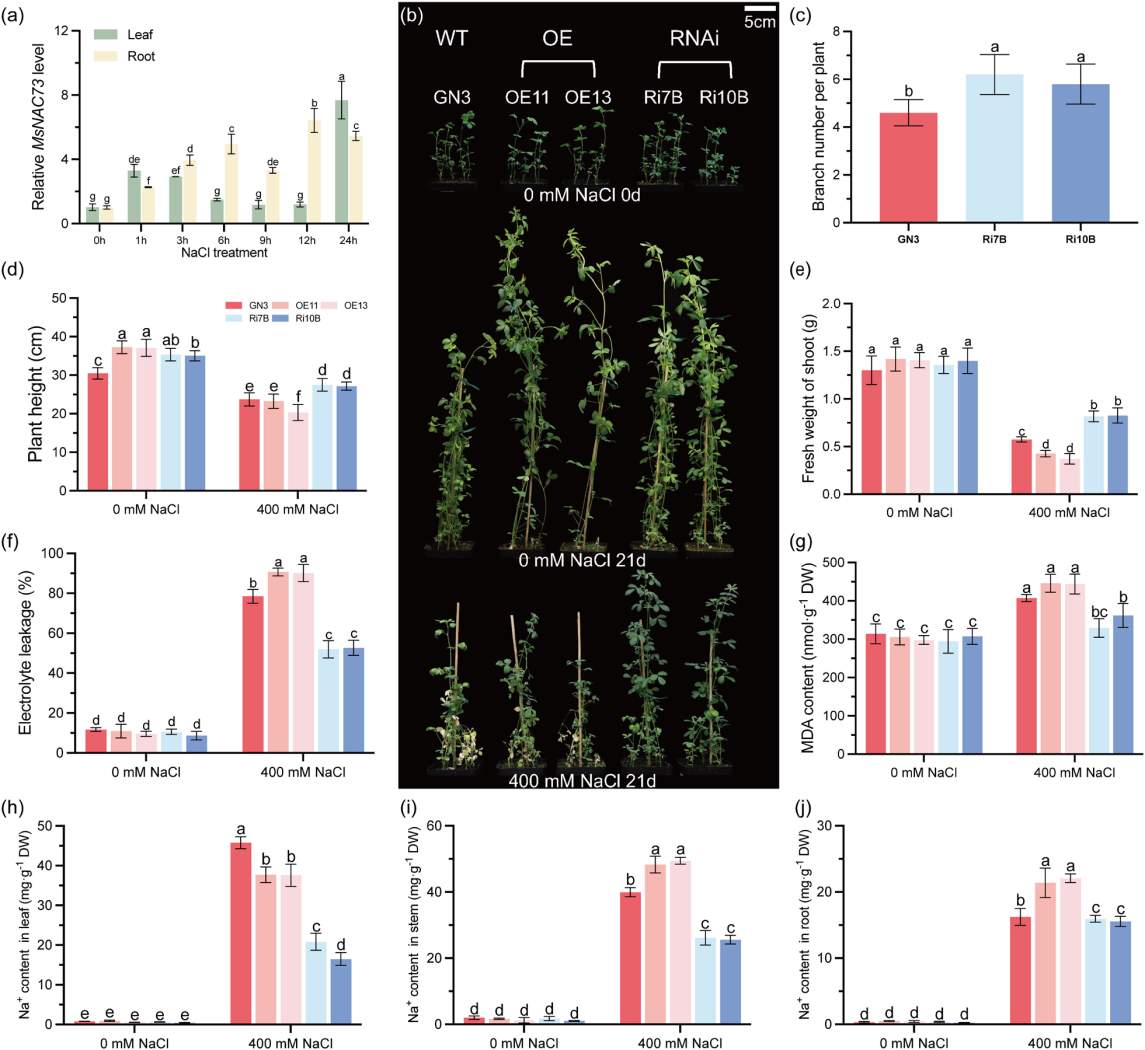
MsNAC73 negatively regulates salt tolerance of alfalfa. (a) The relative expression of *MsNAC73* in leaves and roots of 2‐week‐old alfalfa seedlings treated with 100 mM NaCl at different time points. RNA extracted from leaves and roots was used for RT‐qPCR; *MsEF‐α* and *MsActin2* were used as the internal standards. (b) The uniform wild‐type (WT) and transgenic alfalfa seedlings were grown in vermiculite irrigated with 1/2 Hoagland nutrient solution (pH 5.8), then treated with 0 mM or 400 mM NaCl for 21 days and the phenotypes were recorded. Bar = 5 cm. (c) The branch numbers of WT and *MsNAC73*‐RNAi lines without salt treatment. (d–g) The plant height (d), fresh weight of shoots (e), electrolyte leakage (f) and MDA content (g) of alfalfa treated with 0 or 400 mM NaCl were measured. (h–j) The contents of Na^+^ in leaves (h), stems (i) and roots (j) of alfalfa treated with 0 or 400 mM NaCl were measured. GN3 represents the WT of alfalfa; OE11 and OE13 represent two independent *MsNAC73*‐OE lines; Ri7B and Ri10B represent two independent *MsNAC73*‐RNAi lines. DW, Dry weight; FW, Fresh weight. Data are means ± SE (*n* ≥ 3). Bars with different letters indicate significant differences at *p* < 0.05.

*MsNAC73* expressions were significantly upregulated in leaves and roots of wild‐type (WT) alfalfa seedlings under 100 mM NaCl treatment (Figure 1a). To further elucidate the biological function of *MsNAC73* in response to salt stress, transgenic alfalfa plants were generated through knockdown (RNA interference, RNAi) of *MsNAC73*. PCR amplification of the *kanamycin* resistance genes and RT‐qPCR analysis confirmed that *MsNAC73* was successfully transformed and downexpressed in *MsNAC73*‐RNAi lines (Figure S1). The overexpressing *MsNAC73* (*MsNAC73*‐OE) alfalfa plants had been generated in our earlier study (Fan et al. 2024).

The 3‐week‐old WT and transgenic alfalfa seedlings were treated with 0 mM (normal condition) or 400 mM (salt stress) NaCl for 21 days. Under normal condition, the phenotypes and physiological responses of WT, *MsNAC73*‐OE and *MsNAC73*‐RNAi lines were not significantly different (Figures 1b,d–g and S2), the number of shoot branches significantly increased in *MsNAC73*‐RNAi lines compared to WT plants (Figure 1c). Under salt stress, WT and *MsNAC73*‐OE lines exhibited severe growth retardation and foliar wilting symptoms compared with the *MsNAC73*‐RNAi lines (Figure 1b). The fresh weight of shoots and total chlorophyll contents significantly decreased, while electrolyte leakage (EL) values, malondialdehyde (MDA) contents and H_2_O_2_ accumulation increased dramatically in WT and *MsNAC73*‐OE lines compared to *MsNAC73*‐RNAi lines (Figures 1e–g and S2).

Maintaining Na^+^/K^+^ homeostasis is crucial for plant survival under salt stress conditions (Huang et al. 2024). Under salt stress, *MsNAC73*‐RNAi lines exhibited significantly lower Na^+^ and higher K^+^ contents in leaves, stems and roots compared to WT and *MsNAC73*‐OE lines, while *MsNAC73*‐OE lines showed significantly higher Na^+^ accumulation in stems and roots relative to WT plants (Figures 1h–j and S3a–c), leading to a lower Na^+^/K^+^ ratio in leaves, stems and roots of *MsNAC73*‐RNAi lines than WT and *MsNAC73*‐OE lines, and a higher Na^+^/K^+^ ratio in stems and roots of *MsNAC73*‐OE lines than WT plants (Figure S3d–f).

### 2.2 MsNAC73 Binds to the Promoter of *MsPG2* and Negatively Regulates Its Expression

**FIGURE 2:**
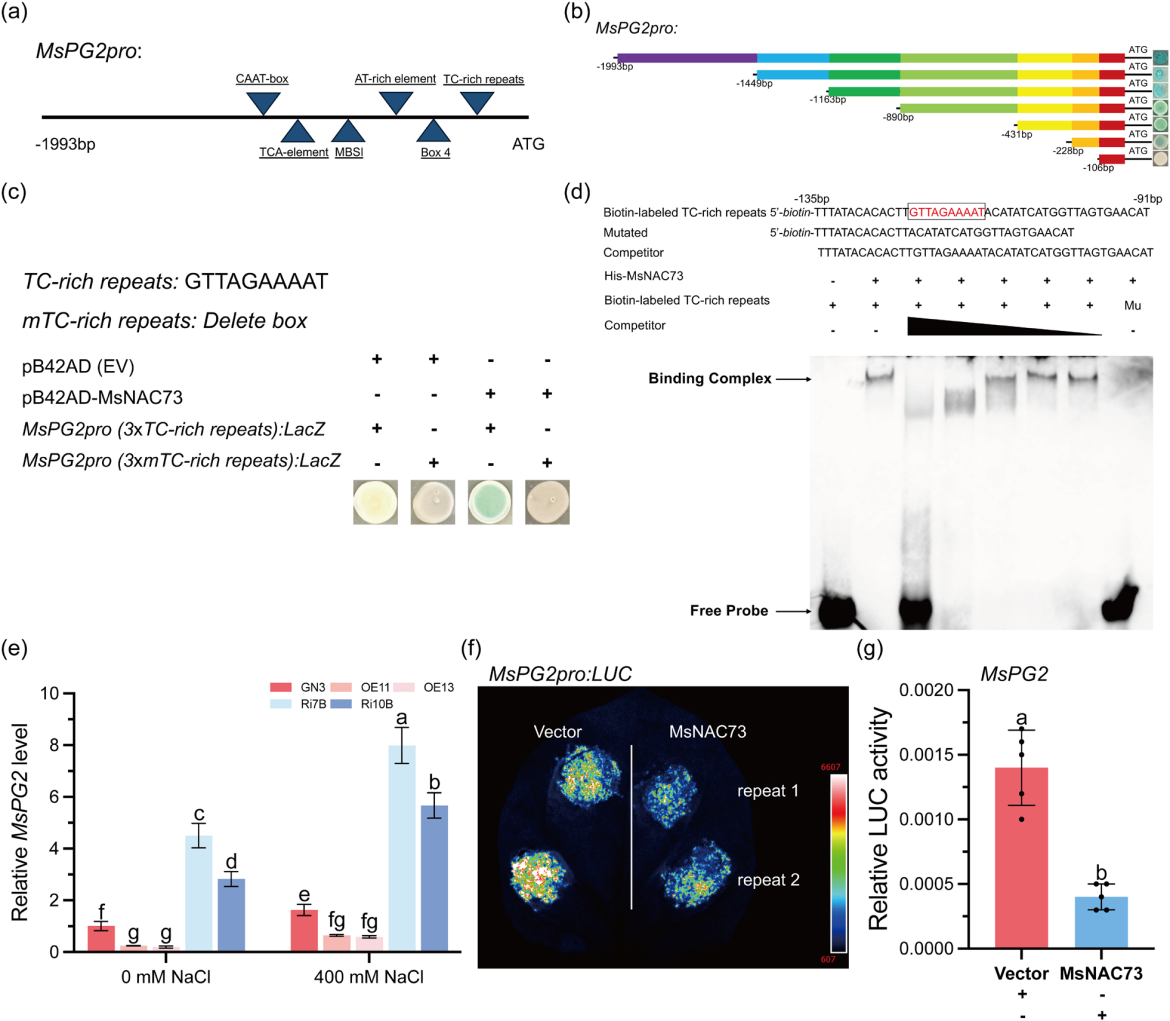
MsNAC73 inhibits the expression of *MsPG2* by directly binding to the TC‐rich repeats in its promoter. (a) The schematic diagram of the *MsPG2* promoter. The predicted motifs are shown as blue triangles. (b) Schematic diagram of *MsPG2* 5′‐promoter deletion constructs; the deletions were ligated into the pLacZ vector and cotransformed with pB42AD‐MsNAC73 into the yeast strain EGY48 for the yeast one‐hybrid (Y1H) assay. The predicted binding sites were located at −228 to −106 bp, which contained TC‐rich repeats. (c) The three tandem copies of the TC‐rich repeats (GTTAGAAAAT) and its mutated motif (mTC‐rich repeats) were synthesised and ligated into the pLacZ vector for the Y1H assay. The pB42AD (empty vector, EV) was used as a negative control. Photos were recorded after 3 days of incubation at 30°C. The assay was repeated three times, and representative results are shown. (d) The EMSA assay showed that MsNAC73 binds to the GTTAGAAAAT (TC‐rich repeats) in the *MsPG2* promoter. Unlabelled TC‐rich repeats were used as a cold competitor. The labelled probe was diluted 20‐fold, and the triangle showed the cold competitors were diluted 1‐, 10‐, 20‐, 50‐ and 100‐fold. Mu, mutated probe in which the TC‐rich repeats were deleted. (e) The relative expression of *MsPG2* in wild‐type (GN3), *MsNAC73*‐OE lines (OE11, OE13) and *MsNAC73*‐RNAi lines (Ri7B, Ri10B) with or without 400 mM NaCl treatment. (f, g) The *MsPG2* promoter driving fluorescence imaging of infected *N. benthamiana* was captured by a living fluorescence imager with D‐luciferin (sodium salt) (f) and the *MsPG2* promoter activities reflected by relative luciferase activities in *N. benthamiana* leaves were simultaneously measured (g). The pHB‐Flag (empty vector) was used as a negative control. Data are means ± SE (*n* ≥ 3). Bars with different letters indicate significant differences at *p* < 0.05.

Our previous and this study have demonstrated that MsNAC73 directly binds to the *MsPAE12* promoter and negatively regulates its expression (Figure S4) (Fan et al. 2024). We inferred that MsNAC73 might affect salt tolerance via modulating the pectins. Thus, the salt treatment section of the MODMS database (a public database of alfalfa, https://modms.lzu.edu.cn) was used to select potential target genes of MsNAC73 (Fang et al. 2023). Firstly, the differentially expressed genes (DEGs) with significant expression differences (≥ 8‐fold or ≤ 0.2‐fold) were screened in the MODMS database based on the *MsNAC73* expression level at each time point. Then, the genes with zero expression were excluded. Finally, using pectin and polygalacturonase as keywords to further screen among the DEGs, 18 DEGs related to pectin hydrolysis and modification were selected as potential target genes of MsNAC73 (Table S1). Subsequently, the promoter regions (~2 kb upstream of the ATG translation initiation site) of these genes were cloned from alfalfa and inserted into the pLacZ vector for Y1H assays. The results showed that MsNAC73 directly binds to the promoter of the polygalacturonase gene named *MsPG2* and activates the *LacZ* reporter gene and *β*‐galactosidase activity (Figure 2).

To further investigate the precise binding sites in the *MsPG2* promoter, the *cis*‐acting regulatory elements were analysed using the plantCARE database (https://bioinformatics.psb.ugent.be/webtools/plantcare/html/). Multiple regulatory elements were observed in the promoter sequence, including CAAT‐box, TCA‐element, MBSI, AT‐rich element, Box 4, TC‐rich repeats and so on (Figure 2a). A series of *MsPG2* 5′‐promoter deletions were ligated into the pLacZ vector and cotransformed with pB42AD‐MsNAC73 into the EGY48 for Y1H assay to further characterise the binding region. The results showed that the potential binding sites were located at −228 to −106 bp, which contained TC‐rich repeats (Figure 2b). Subsequently, the Y1H assay demonstrated that pB42AD‐MsNAC73 specifically bound to three tandem copies of the TC‐rich repeats (GTTAGAAAAT) within the *MsPG2* promoter. In contrast, no binding was observed in the mutated motif (mTC‐rich repeats) (Figure 2c). Electrophoretic mobility shift assays (EMSAs) were conducted to confirm these results in vitro. A single protein‐DNA complex band was detected when His‐NAC73 was incubated with labelled DNA probes containing the TC‐rich repeats. The interactions were weakened when the unlabelled TC‐rich repeats competitors with the same sequence were added in excess. No binding band was observed when the TC‐rich repeats were deleted (Mu) or when the labelled DNA probe was present alone (Figure 2d). These results proved that MsNAC73 directly binds to the TC‐rich repeats in the *MsPG2* promoter.

The transcriptional regulation of MsNAC73 on *MsPG2* expression was further investigated through RT‐qPCR analysis in transgenic lines. The expression of *MsPG2* significantly downregulated in *MsNAC73*‐OE lines and upregulated in *MsNAC73*‐RNAi lines compared to WT plants under normal and salt stress conditions (Figure 2e). In addition, the dual‐LUC assays were performed in tobacco (*Nicotiana benthamiana*) leaves by cotransforming *35S::Flag*/*35S::MsNAC73‐Flag* effector and *MsPG2pro:LUC* reporter. MsNAC73 significantly attenuated the reporter, which exhibited luciferase activities 3.5‐fold lower than that with *35S::Flag* as the effector (Figure 2f,g). These results indicated that MsNAC73 negatively regulates the *MsPG2* expression.

### 2.3 *MsPG2* Highly Expresses in Stems and Localises on the Cell Wall

The protein conserved domain prediction revealed that MsPG2 had a polysaccharide lyase family 6 (PL‐6) region and contained four conserved residues of the PG proteins. Sequence alignment demonstrated that MsPG2 shared 92.7% of sequence identity with AES59005.1 from *Medicago truncatula* , which has been defined as a polygalacturonase (Figure S5a,b). The phylogenetic tree was constructed using the PG sequences from *M. truncatula* , *A. thaliana* and three previously reported MsPGs from *M. sativa* (Fan et al. 2022; Li et al. 2020; Muñoz et al. 1998). The phylogenetic analysis suggested that MsPG2 belongs to Clade F and has a high homology to AES59005.1 (Figure S5c).

Tissue‐specific expression analysis via RT‐qPCR revealed that *MsPG2* expressed in a wide range of tissues with predominant expression in stems (52‐fold higher than buds) (Figure S6a). To investigate the subcellular localisation of MsPG2, the *Agrobacterium tumefaciens* strain GV3101 harbouring the 35S::MsPG2‐YFP fusion construct was transiently transformed into *N. benthamiana* and epidermal cells of onion ( *Allium cepa* ). Confocal microscopy observation revealed that MsPG2 localised at the cell periphery. Subsequent plasmolysis analysis showed that the YFP fluorescence signal remained at the cell periphery when the plasma membrane retracted, confirming that MsPG2 localises on the cell wall (Figure S6b).

### 2.4 MsPG2 Contributes to the Salt Tolerance of Alfalfa

RT‐qPCR assay revealed that the expressions of *MsPG2* in both leaves and roots of WT alfalfa seedlings were significantly induced by 100 mM NaCl treatment (Figure S6c). To further investigate the underlying molecular mechanisms of *MsPG2* response to salt stress, transgenic alfalfa lines of overexpression (OE) and knockdown (RNA interference, RNAi) of *MsPG2* were generated via *Agrobacterium*‐mediated transformation (Figure S7).

**FIGURE 3:**
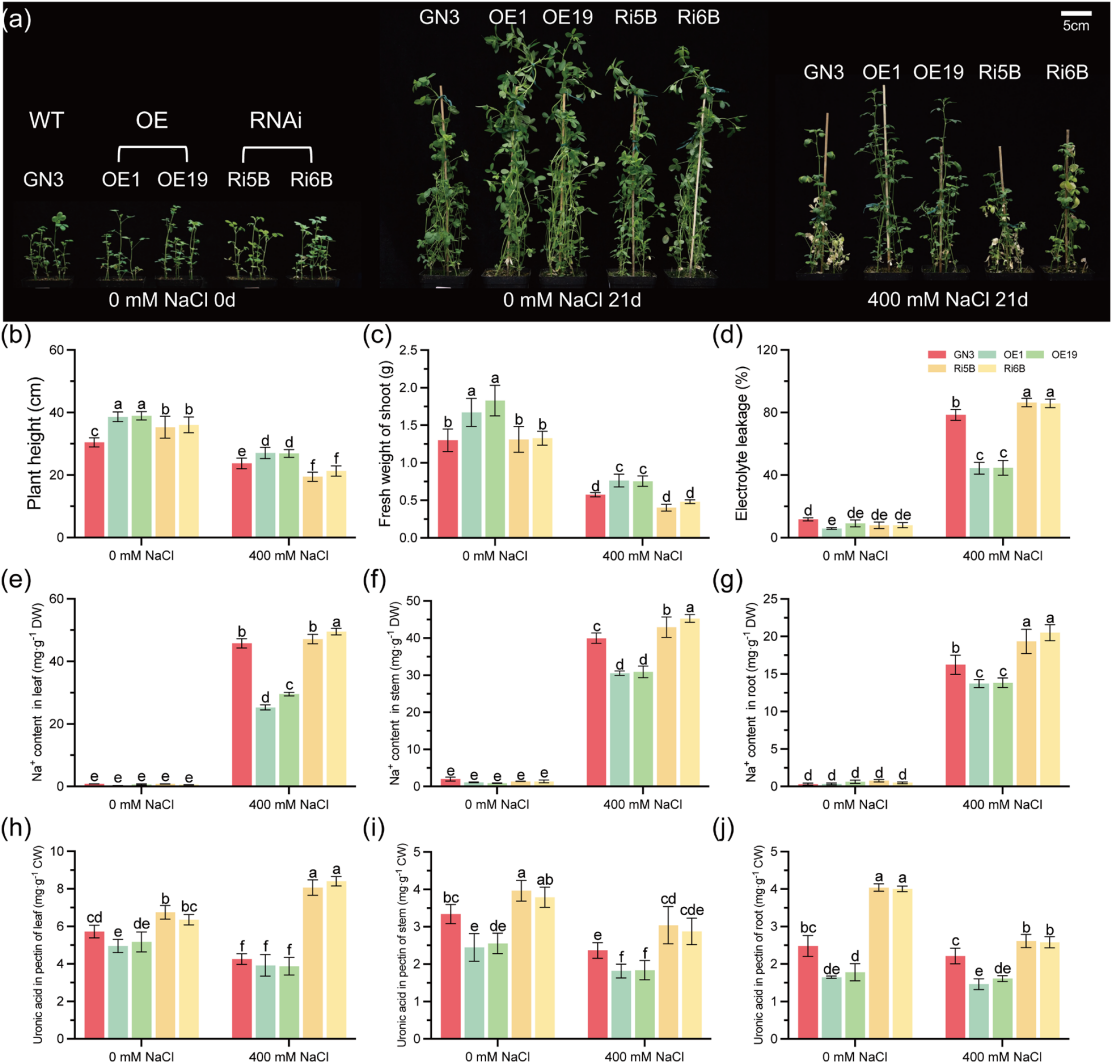
Overexpression of *MsPG2* enhances salt tolerance of alfalfa. (a) The uniform wild‐type (WT) and transgenic alfalfa were grown in vermiculite irrigated with 1/2 Hoagland nutrient solution (pH 5.8), then treated with 0 mM or 400 mM NaCl for 21 days and the phenotypes were recorded. Bar = 5 cm. (b–d) The plant height (b), fresh weight of shoots (c) and electrolyte leakage (d) of alfalfa seedlings treated with 0 or 400 mM NaCl were measured. (e–g) The contents of Na^+^ in leaves (e), stems (f) and roots (g) of alfalfa treated with 0 or 400 mM NaCl were measured. (h–j) The uronic acid content in pectins extracted from leaves (h), stems (i) and roots (j) of alfalfa treated with 0 or 400 mM NaCl. GN3 represents the WT of alfalfa; OE1 and OE19 represent two independent *MsPG2*‐OE lines; Ri5B and Ri6B represent two independent *MsPG2*‐RNAi lines. CW, Cell wall; DW, Dry weight. Data are means ± SE (*n* ≥ 3). Bars with different letters indicate significant differences at *p* < 0.05.

Under normal and salt stress conditions, *MsPG2*‐OE lines had significantly higher height and fresh weight of shoots than WT and *MsPG2*‐RNAi lines (Figure 3a–c). In the absence of NaCl treatment, no statistically significant differences were observed among WT and transgenic alfalfa lines regarding total chlorophyll content, EL value and the contents of MDA and H_2_O_2_. When exposed to 400 mM NaCl stress, however, the WT and *MsPG2*‐RNAi lines exhibited more serious damage than *MsPG2*‐OE lines, with significantly lower chlorophyll content, higher EL values and MDA and H_2_O_2_ contents than *MsPG2*‐OE lines, while *MsPG2*‐RNAi lines suffered more serious damage than WT plants (Figures 3d and S8). Under salt stress, Na^+^ contents in leaves, stems and roots of *MsPG2*‐OE lines were significantly lower than WT and *MsPG2*‐RNAi lines, while K^+^ content in the three tissues of *MsPG2*‐OE lines sustained a high level, leading to a lower Na^+^/K^+^ ratio, decreased by 61.7%, 32.1% and 24.4% in the leaves, stems and roots of *MsPG2*‐OE lines than WT plants respectively. While the *MsPG2*‐RNAi lines showed higher Na^+^/K^+^ ratios than WT plants (Figures 3e–g and S9). These results suggested that *MsPG2* expression positively affects the salt tolerance of alfalfa.

### 2.5 MsPG2 Enhances Salt Tolerance of Alfalfa via Mediating Pectin Hydrolysis and Na^+^/K^+^ Homeostasis in the Cell Wall

The pectin contents in the cell walls of stems and roots significantly decreased in *MsPG2*‐OE lines, but increased in *MsPG2*‐RNAi lines compared with WT plants under normal and salt stress conditions (Figure 3h–j), which was contrary to the pectin contents in the cell walls of *MsNAC73*‐OE and *MsNAC73*‐RNAi lines respectively (Figure S10). While no significant difference in hemicellulose 1 contents in the cell walls of leaves, stems and roots was observed among the WT plants, *MsPG2*‐OE and *MsPG2*‐RNAi lines (Figure S11).

To investigate the effect of pectins on ion accumulation in the cell wall, K^+^ and Na^+^ contents in the cell walls of leaves, stems and roots were measured. In the absence of salt, the K^+^ contents in the cell wall of the three tissues were higher in *MsPG2*‐RNAi lines than that of the WT and *MsPG2*‐OE lines; however, there were no significant differences under salt stress, suggesting that the K^+^ adsorption capacity of the cell wall positively correlated with the pectin content under normal conditions, but salt stress disturbed the K^+^ homeostasis in the cell wall (Figure S12a–c). Under salt stress, Na^+^ accumulation in the cell wall of the three tissues was significantly higher in the *MsPG2*‐RNAi lines, but lower in *MsPG2*‐OE lines compared to WT plants (Figure S12d–f). The Na^+^/K^+^ ratio in the cell walls of leaves, stems and roots showed similar results to the Na^+^ content in the WT and transgenic plants (Figure S12g–i). These results were consistent with Na^+^ accumulation observed in the three tissues of WT and transgenic plants, indicating that pectin content in the cell wall directly determines the absorption and accumulation of Na^+^ in plants via modulating the Na^+^/K^+^ ratio and affects plant tolerance to salt stress.

### 2.6 *MsPG2* Expression Positively Regulates Leaf Stomatal Aperture and Xylem Vessel Diameter of Stem

**FIGURE 4:**
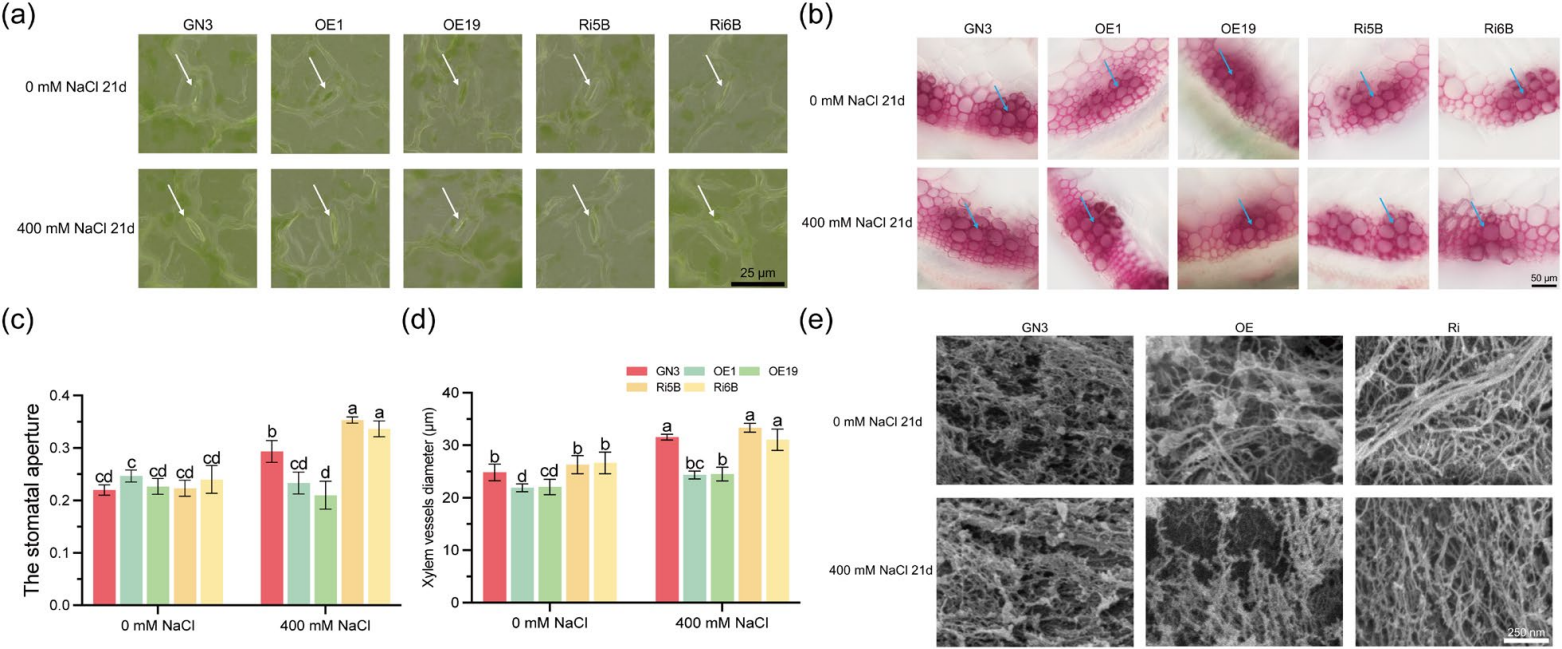
MsPG2 modulates stomata, xylem vessels and cell wall structure of alfalfa. (a–d) The microscope images of stomata (a, white arrowheads) and xylem vessels of stems (b, blue arrowheads), the quantification of stomatal aperture (c) and the diameter length of xylem vessels (d) in wild‐type (WT) and transgenic alfalfa treated with 0 mM or 400 mM NaCl for 21 days. The stomatal aperture = stomatal width/stomatal length, and the unit is ‘1’. Bar = 25 μm. The fifth internode of stems was cut by freehand sectioning and stained with phloroglucinol. Bar = 50 μm. Data are means ± SE (*n* ≥ 3). Bars with different letters indicate significant differences at *p* < 0.05. (e) The microstructures of cell walls on the root tips' surface were observed by Raman image‐scanning electron microscopy (RISE‐MAGNA), with or without 400 mM NaCl. Bar = 250 nm. GN3 represents the WT of alfalfa; OE1 and OE19 represent two independent *MsPG2*‐OE lines; Ri5B and Ri6B represent two independent *MsPG2*‐RNAi lines.

In order to investigate the effect of MsPG2 on the driving force of Na^+^ transport from roots to shoots, the stomatal aperture of leaf epidermises and the xylem vessel diameter of the stem were analysed. Under salt stress, the stomatal aperture was decreased and smaller in *MsPG2*‐OE lines, but increased and larger in WT and *MsPG2*‐RNAi lines compared to normal condition, whereas no significant difference was observed under normal condition (Figure 4a,c). The vessel diameter in *MsPG2*‐OE lines was significantly reduced compared to WT and *MsPG2*‐RNAi lines with or without salt stress (Figure 4b,d), indicating that MsPG2 effectively attenuates the driving force of Na^+^ transport from roots to shoots under salt stress.

To further investigate the effect of MsPG2 on the cell wall structure, the microstructures of the cell wall were observed by RISE‐MAGNA. Salt stress caused the organisation of cellulose microfibrils to be denser with smaller porosities of cross‐linked cellulose microfibrils, whereas *MsPG2*‐OE lines maintained a relatively loose cell wall structure with larger porosity, and *MsPG2*‐RNAi lines showed a relatively dense cell wall structure with smaller porosity compared with WT plants (Figure 4e). These observations suggest that overexpression of *MsPG2* effectively sustains a loose cell wall structure and increases the cell wall extensibility under salt stress.

### 2.7 The MsNAC73 Interacts With a Mitogen‐Activated Protein Kinase MsMPK3

To investigate the molecular mechanism of MsNAC73 that regulates the salt stress response of alfalfa, a pull‐down assay coupled with mass spectrometry analysis was performed to screen the interacting proteins of MsNAC73 (Figure S13). One hundred and sixty‐five potential MsNAC73‐interacting proteins were found, including six protein kinases (Table S2). Concurrently, potential phosphorylation sites within MsNAC73 were predicted using GPS 6.0 (http://gps.biocuckoo.cn) (Table S3). Further validation of the interactions between MsNAC73 and each candidate protein kinase was systematically conducted.

**FIGURE 5:**
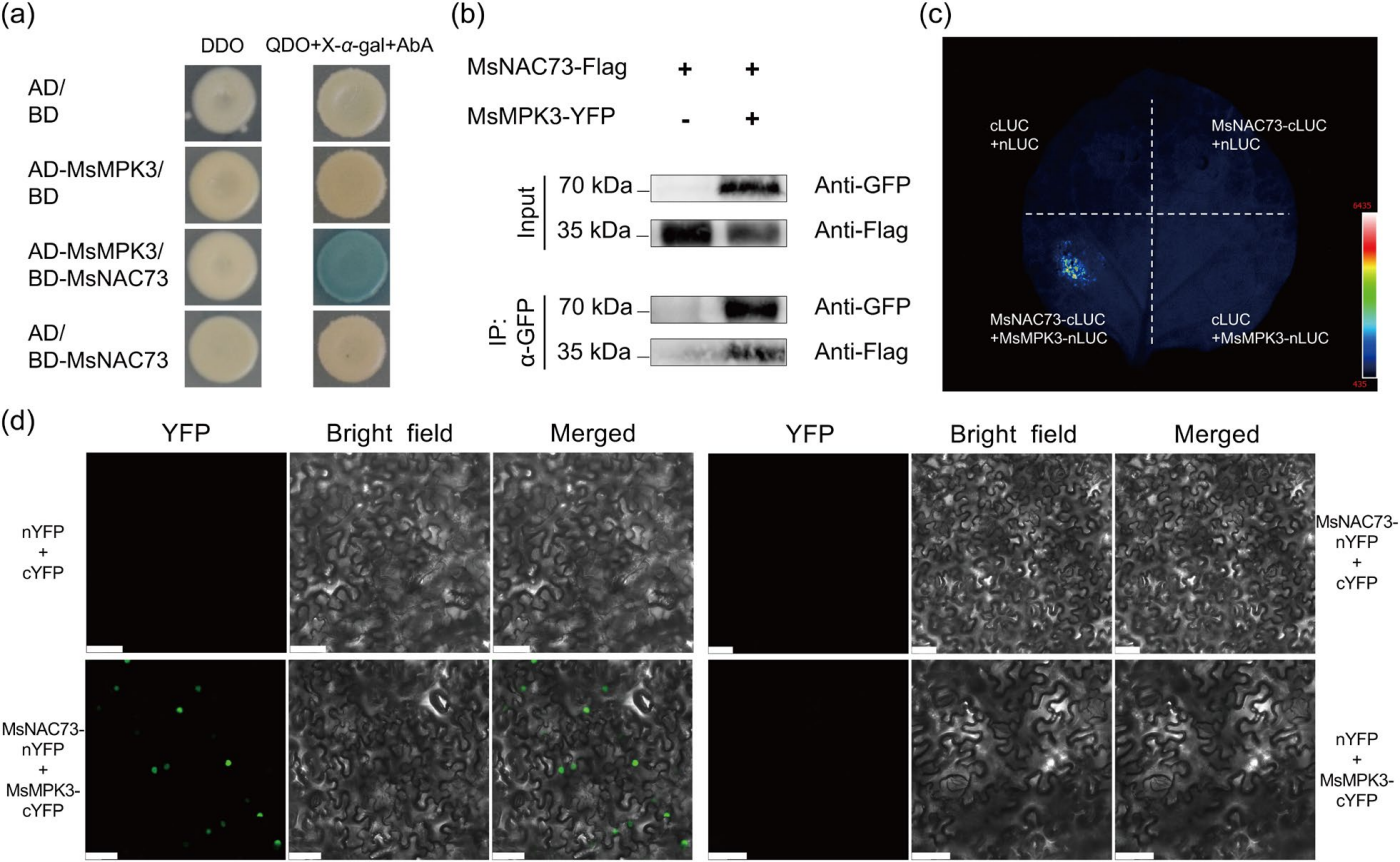
MsNAC73 interacts with MsMPK3 in vivo. (a) The yeast two‐hybrid (Y2H) assay demonstrated the interaction between MsNAC73 and MsMPK3. Yeast cells were grown on the selective media, DDO and QDO + X‐*α*‐Gal + AbA. Photos were recorded after 3 days of incubation at 30°C. (b) The coimmunoprecipitation (Co‐IP) assay indicated the interactions between MsNAC73 and MsMPK3. Co‐IP was conducted with anti‐GFP nanobody agarose beads, and anti‐GFP and anti‐Flag antibodies were used to detect MsMPK3 and MsNAC73 respectively. (c) The split‐LUC assay validated the interactions between MsNAC73 and MsMPK3. The fluorescence imaging of infected *N. benthamiana* was captured by a living fluorescence imager with D‐luciferin (sodium salt). (d) Bimolecular fluorescence complementation (BiFC) assay showed the interaction between MsNAC73 and MsMPK3. The MsNAC73 and MsMPK3 were fused to the N‐terminal fragment (nYFP) or C‐terminal fragment (cYFP) of YFP respectively. Bar = 50 μm. All analyses were repeated three times independently, and representative results are shown.

The yeast two‐hybrid (Y2H) assay revealed that a mitogen‐activated protein kinase designated MsMPK3 was identified as a potential interactor. When cotransformed AD‐MsMPK3 with BD‐MsNAC73, the enabled yeast cells grew and turned blue on QDO (SD/−Trp/−Leu/‐His/−Ade) + X‐*α*‐Gal + AbA selective medium (Figure 5a). The MsNAC73‐MsMPK3 interaction was also corroborated through coimmunoprecipitation (Co‐IP) assays (Figure 5b). Furthermore, the split luciferase complementation assay provided additional evidence confirming this protein–protein interaction (Figure 5c). Finally, bimolecular fluorescence complementation (BiFC) assays were performed to demonstrate MsNAC73–MsMPK3 interaction. When MsNAC73‐nYFP (N‐terminal fragment of YFP) was transiently coexpressed with MsMPK3‐cYFP (C‐terminal fragment of YFP) in *N. benthamiana* epidermal cells, reconstituted YFP fluorescence signals were observed in the nucleus. At the same time, negative controls exhibited no detectable YFP fluorescence (Figure 5d).

### 2.8 Salt‐Induced Increase of MsMPK3 Kinase Activity Mediates Phosphorylation of MsNAC73

**FIGURE 6:**
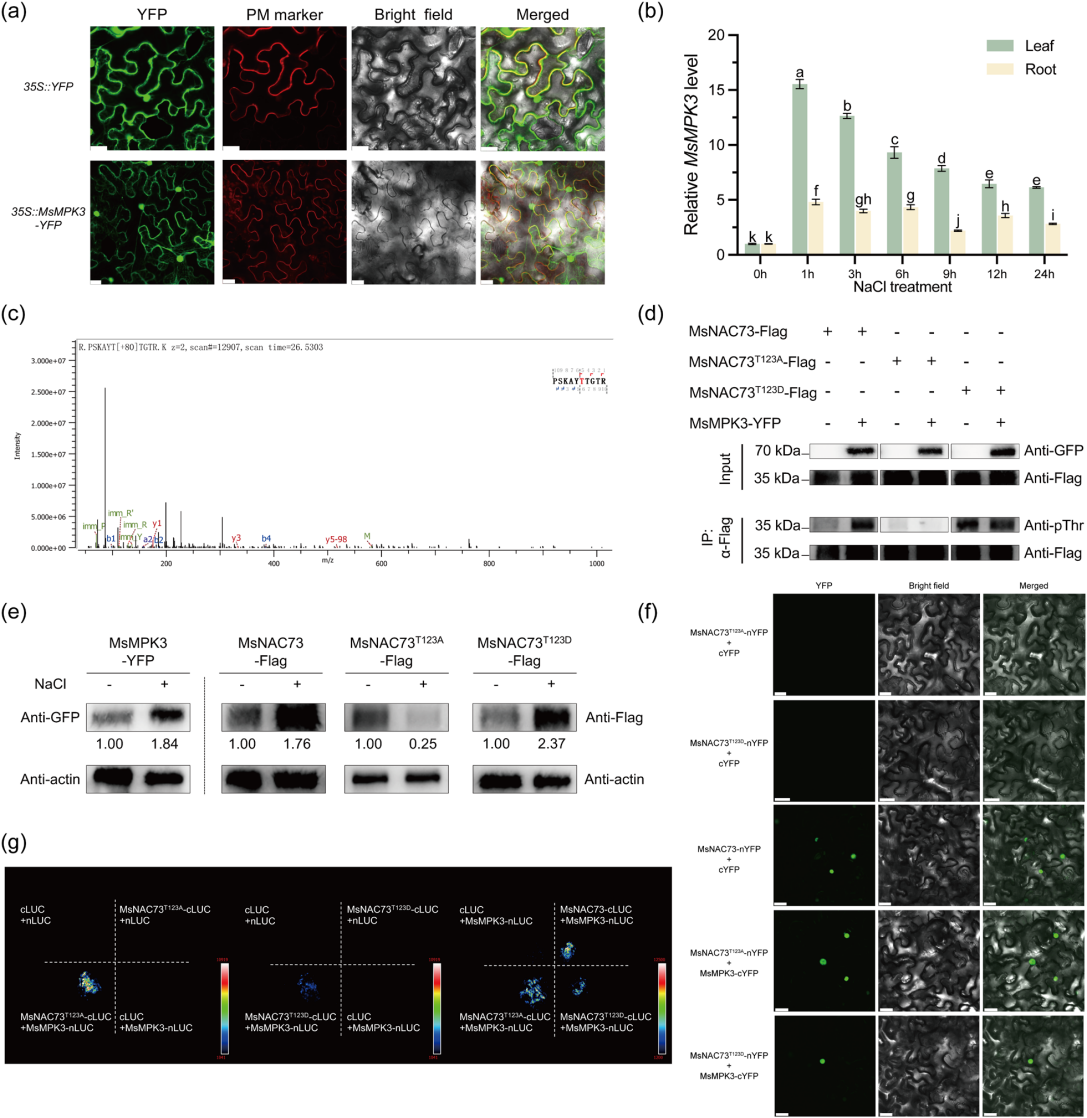
MsMPK3 phosphorylates MsNAC73 at Thr‐123. (a) The subcellular localisation of MsMPK3 in *N. benthamiana* leaves. The 35S::MsMPK3‐YFP and plasma membrane marker (CD3‐1007; PM‐marker) were coinfiltrated into *N. benthamiana* leaves; the fluorescent signals were observed using confocal microscopy after 48 h of incubation. 35S::YFP was used as a control. Bar = 25 μm. (b) The relative expressions of *MsMPK3* in 2‐week‐old leaves and roots of alfalfa seedlings treated with 100 mM NaCl at different time points. RNA extracted from leaves and roots was used for RT‐qPCR; *MsEF‐α* and *MsActin2* were used as the internal standards. Data are means ± SE (*n* ≥ 3). Bars with different letters indicate significant differences at *p* < 0.05. (c) The phosphorylation sites of MsNAC73 were detected by LC–MS/MS. (d) The Thr‐123 residue is the primary MsMPK3 phosphorylation site in MsNAC73. The coimmunoprecipitation (Co‐IP) assay indicated that mutation of Thr‐123 in MsNAC73 blocked its phosphorylation by MsMPK3. Co‐IP was conducted with anti‐Flag nanobody agarose beads, and anti‐GFP antibody was used to detect MsMPK3. The anti‐pThr and anti‐Flag antibodies were used to detect MsNAC73, MsNAC73^T123A^ and MsNAC73^T123D^. (e) Western blotting analyses were performed using anti‐GFP or anti‐Flag antibodies to detect the abundance of MsMPK3, MsNAC73, MsNAC73^T123A^ and MsNAC73^T123D^ in *N. benthamiana* treated with or without salt. Actin bands detected using antiactin antibody indicate protein loading. +: Treated with 200 mM NaCl for 24 h; −: treated with 0 mM NaCl for 24 h. (f) Bimolecular fluorescence complementation (BiFC) assay showed the interaction between MsMPK3 and MsNAC73, MsNAC73^T123A^, or MsNAC73^T123D^. The MsNAC73, MsNAC73^T123A^ and MsNAC73^T123D^ were fused to the N‐terminal fragment (nYFP) of YFP, and the MsMPK3 was fused to the C‐terminal fragment (cYFP) of YFP respectively. Bar = 30 μm. (g) The split‐LUC assay validated the interactions between MsMPK3 and MsNAC73, MsNAC73^T123A^ or MsNAC73^T123D^. The fluorescence imaging of infected *N. benthamiana* was captured by a living fluorescence imager with D‐luciferin (sodium salt). T: Thr; D: Asp; A: Ala; MsNAC73^T123D^: Phosphomimetic form; MsNAC73^T123A^: Phosphorylation‐deficient form. All analyses were repeated three times independently, and representative results are shown.

Subcellular localisation analyses showed that MsMPK3 localised on the cell membrane, cytoplasm and nucleus (Figure 6a). Our previous study demonstrated that MsNAC73 is localised in the nucleus (Fan et al. 2024), verifying that the two proteins colocalised in the nucleus. The expression of *MsMPK3* was notably induced by 100 mM NaCl treatment in both leaves and roots (Figure 6b).

The liquid chromatography–mass spectrometry (LC–MS/MS) was conducted to identify the phosphorylation sites in MsNAC73. The analysis revealed that the Thr‐123, Ser‐245 or Ser‐22 residues in MsNAC73 were phosphorylated by MsMPK3 (Figures 6c, S14 and Table S4). Based on previous findings that the Thr‐26 residue in ZmNAC49 serves as the primary site phosphorylated by ZmMPK5 (Xiang et al. 2021), the Thr‐123 residue was selected to validate whether it was the primary phosphorylation site of MsNAC73. The phosphomimetic and phosphorylation‐deficient forms of MsNAC73 were generated by introducing mutations of Thr‐123 to either Asp (T123D) or Ala (T123A) respectively. Co‐IP assays revealed that MsMPK3‐YFP strongly phosphorylated MsNAC73‐Flag detected by the anti‐pThr antibody; similar results were observed in the treatments of MsNAC73^T123D^‐Flag and MsMPK3‐YFP phosphorylated MsNAC73^123D^‐Flag. In contrast, the phosphorylation of MsNAC73 by MsMPK3 was largely blocked when Thr‐123 was mutated to Ala in MsNAC73 (MsNAC73^T123A^‐Flag) (Figure 6d). These results suggested that the Thr‐123 residue in MsNAC73 is the principal site phosphorylated by MsMPK3.

Furthermore, Western blotting analyses revealed that the protein abundance of MsMPK3, MsNAC73 and the phosphomimetic form MsNAC73^T123D^ was significantly elevated in *N. benthamiana* leaves under salt stress conditions compared to normal conditions. Conversely, the phosphorylation‐deficient form MsNAC73^T123A^ exhibited a marked reduction in protein abundance under salt treatment (Figure 6e). To further investigate whether phosphorylation of MsNAC73 affected its interaction with MsMPK3, both BiFC and the split luciferase complementation assay were performed. Compared to MsNAC73, the phosphomimetic form MsNAC73^T123D^ significantly impaired its interaction with MsMPK3, reflected by fewer green fluorescence signals (Figure 6f) and weaker LUC activity levels (Figure 6g) than those of the interaction of MsNAC73 and MsMPK3, while the phosphorylation‐deficient form MsNAC73^T123A^ did not affect its interaction with MsMPK3.

### 2.9 The Differential Phosphorylation Status of MsNAC73 Regulates the Expression of *MsPG2* and *MsPAE12* to Balance Shoot Branching and Salt Tolerance

**FIGURE 7:**
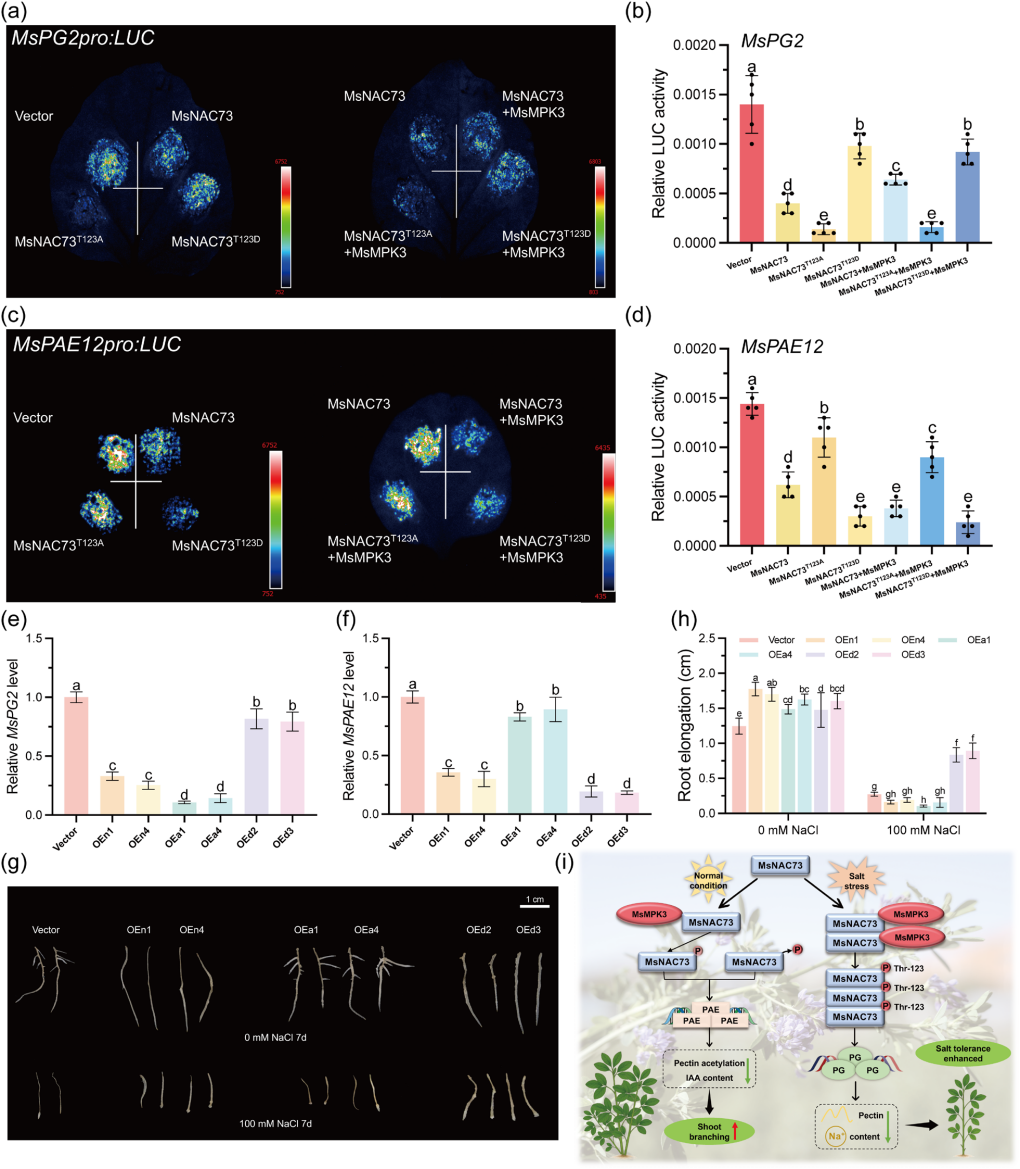
The MsNAC73‐MsMPK3 module regulates the expression of *MsPG2* and *MsPAE12*. (a–d) The expressions and activities of *MsPG2pro:LUC* (a, b) and *MsPAE12 pro:LUC* (c, d) were regulated by MsNAC73, MsNAC73^T123A^ and MsNAC73^T123D^ independently or combined with MsMPK3. The fluorescence imaging of infected *N. benthamiana* was captured by a living fluorescence imager with D‐luciferin (sodium salt), and the relative luciferase activities in *N. benthamiana* leaves were simultaneously measured. The pHB‐Flag (empty vector) was used as a negative control. (e, f) The relative expressions of *MsPG2* (e) and *MsPAE12* (f) in transgenic alfalfa hairy roots. (g, h) The phenotype (g) and root length (h) of transgenic alfalfa hairy roots treated with 0 or 100 mM NaCl for 7 days. Bar = 1 cm. The vector represents the control line; OEn1 and OEn4 represent two independent *MsNAC73*‐OE lines; OEa1 and OEa4 represent two independent *MsNAC73* ^*T123A*^‐OE lines (phosphorylation‐deficient form); OEd2 and OEd3 represent two independent *MsNAC73* ^*T123D*^‐OE lines (phosphomimetic form). Data are means ± SE (*n* ≥ 3). Bars with different letters indicate significant differences at *p* < 0.05. (i) A proposed model of MsNAC73‐MsMPK3‐*MsPAE12/MsPG2* coinfluencing shoot branching and salt tolerance of alfalfa. Under normal conditions, the low protein abundance of MsMPK3 and MsNAC73 causes their weak interaction and diminishes MsNAC73 phosphorylation, thereby attenuating the transcriptional repression of MsNAC73 to *MsPAE12* expression. The upregulation of *MsPAE12* increases shoot branching through reducing pectin acetylation and IAA biosynthesis. Under salt stress conditions, the abundance of MsMPK3 and MsNAC73 increases, and their interaction and the phosphorylation of MsNAC73 are strengthened, consequently attenuating the transcriptional repression of MsNAC73 to *MsPG2* expression. The upregulation of *MsPG2* promotes pectin hydrolysis, leading to increases in cell wall extensibility and Na^+^/K^+^ homeostasis, and reduction of stomatal aperture and vessel diameter, thereby enhancing the salt tolerance of alfalfa.

Our previous study reported that MsNAC73 negatively regulates the expression of *MsPAE12*, which mediates the shoot branching of alfalfa by affecting IAA biosynthesis (Fan et al. 2024). To further investigate the regulatory mechanism underlying the MsNAC73 phosphorylation on its two downstream target gene expressions of *MsPG2* and *MsPAE12*, the dual‐LUC assays were performed in *N. benthamiana* leaves by cotransforming *35S::Flag* (empty vector) or *35S::MsNAC73‐Flag/35S::MsNAC73* ^*T123A*^ *‐Flag/35S::MsNAC73* ^*T123D*^ *‐Flag* either independently or coexpressed with *35S::MsMPK3‐YFP* effector and *MsPG2pro:LUC/MsPAE12pro:LUC* reporter (Figure 7a–d). The transient expression assays revealed that coexpression of MsNAC73 with the *MsPG2pro:LUC* reporter significantly attenuated LUC signals relative to the empty vector (35S::Flag), while the phosphorylation‐deficient form MsNAC73^T123A^ promoted the repressive effect on LUC signals. In contrast, the phosphomimetic form MsNAC73^T123D^ diminished the repressive effect, with similar luciferase signals to the empty vector and a stronger signal than MsNAC73^T123A^ (Figure 7a). Further, the MsMPK3‐mediated phosphorylation (MsNAC73 + MsMPK3 and MsNAC73^T123D^ + MsMPK3) significantly enhanced luciferase signals compared to only MsNAC73 expressed. Conversely, coexpression of MsNAC73^T123A^ with MsMPK3 maintained robust repression of LUC signals (Figure 7a). These results were further confirmed by the *MsPG2pro* driven LUC activities, which showed similar results with LUC signals under the above treatments (Figure 7b).

For the regulation of *MsPAE12* expression, when the *MsPAE12* promoter fused with LUC and MsNAC73 was coexpressed in *N. benthamiana* leaves, the luciferase signals (Figure 7c) and activities (Figure 7d) were attenuated compared with the empty vector (35S::Flag), but the luciferase signals and activities were significantly enhanced when the *MsPAE12pro:LUC* and the phosphorylation‐deficient form (MsNAC73^T123A^ or MsNAC73^T123A^ + MsMPK3) were coexpressed relative to only MsNAC73 expressed. Conversely, both the MsMPK3‐mediated MsNAC73 phosphorylation (MsNAC73 + MsMPK3) and phosphomimetic form (MsNAC73^T123D^ and MsNAC73^T123D^ + MsMPK3) showed significantly reduced LUC signals and activities (Figure 7c,d).

To validate the above findings, we generated transgenic alfalfa hairy roots through overexpression (OE) of *MsNAC73*, *MsNAC73* ^*T123A*^ and *MsNAC73* ^*T123D*^ (Figure S15). RT‐qPCR analysis revealed that overexpressing *MsNAC73* (OEn1 and OEn4) significantly inhibited the expressions of *MsPG2* and *MsPAE12* in hairy roots compared with the vector line (Figure 7e,f), corresponding with the results of *MsPG2* and *MsPAE12* expressions in *MsNAC73*‐OE and ‐RNAi transgenic alfalfa plants (Figures 2e and S4). Interestingly, *MsPG2* expression was further inhibited in the *MsNAC73* ^*T123A*^‐OE lines (OEa1 and OEa4), whereas *MsPAE12* expression was significantly upregulated in the *MsNAC73* ^*T123A*^‐OE lines compared with the *MsNAC73*‐OE lines. In contrast, *MsPG2* expression was significantly upregulated in the *MsNAC73* ^*T123D*^‐OE lines (OEd2 and OEd3), while *MsPAE12* expression was significantly inhibited in the *MsNAC73* ^*T123D*^‐OE lines compared with the *MsNAC73*‐OE lines. These results indicate that MsMPK3‐mediated MsNAC73 phosphorylation positively and negatively regulates the expressions of *MsPG2* and *MsPAE12* respectively.

Phenotype analysis showed that the *MsNAC73* ^*T123A*^‐OE lines displayed a significant increase in lateral root number under normal conditions, while the *MsNAC73* ^*T123D*^‐OE lines exhibited a significantly higher root elongation than other lines under salt stress (Figures 7g,h and S16). To sum up, the above results suggested that phosphorylation and dephosphorylation of MsNAC73 regulate the expressions of *MsPG2* and *MsPAE12* respectively, to balance salt response and shoot branching.

## 3 Discussion

### 3.1 *MsPG2* Expression Positively Affects Alfalfa Tolerance to NaCl Stress via Hydrolysing Pectins and Regulating Na^+^/K^+^ Homeostasis in Plants

Polygalacturonases (PGs), the key pectin hydrolases in plants, are widely expressed in various plant tissues and play essential roles in modulating pectin degradation and pectin‐affected cell elongation and adhesion (Jiang et al. 2019; Wu et al. 2020; Xiao et al. 2017, 2014). The increased polygalacturonase activities in overexpressing *PG* plants degrade the pectin contents, shorten the homogalacturonan chains, attenuate the calcium‐cross‐linked pectin network, consequently causing a loose cell wall structure, large cell wall expansibility, intercellular gaps and cell elongation (Cosgrove 1999; Du et al. 2022; Phyo et al. 2017). The effects of PGs on plant responses to cold and drought stresses have been well studied (He et al. 2023; Page et al. 2010; Zhang, Hou, et al. 2021); however, the PGs are overlooked in studying the plant response to salt stress. In the present study, the *MsPG2* is highly expressed in the cell wall of stems, similar to previous findings (Xiao et al. 2017; Yang et al. 2021). Salt stress upregulated *MsPG2* expression, and the salt tolerance of transgenic alfalfa increased in *MsPG2*‐OE lines, but decreased in *MsPG2*‐RNAi lines, indicating that *MsPG2* expression positively affects alfalfa tolerance to salt stress. Overexpression of *MsPG2* in alfalfa promoted pectin hydrolysis, decreased pectin content in cell walls and sustained a loose cellulose microfibril structure under salt stress, which increased cell wall extensibility of salt‐stressed *MsPG2*‐OE lines. These results are consistent with our previous studies that overexpression of *MsPG1* or *MsPG4* in alfalfa effectively reduces pectin contents in the cell walls of root tips, leading to decreased Al accumulation and increased cell wall extensibility in root tips, and mitigates Al^3+^ toxicity (Fan et al. 2022; Li et al. 2020). Thus, increased cell wall extensibility modulated by pectin hydrolysis can effectively improve plant tolerance to salt stress.

Pectins can affect Na^+^ accumulation in the cell wall, which serves as the first barrier to prevent the entry of Na^+^ into the cell, and then affect the salt tolerance of plants (Colin et al. 2023; Dabravolski and Isayenkov 2023). Pectin demethylation directly causes pectin with negative charges, which leads to Na^+^ retention within the cell wall of oilseed rape ( *Brassica napus* ); in addition, mutation of *OsTSD2*, which encodes pectin methyltransferase, leads to the downregulation of key ion homeostasis‐related genes (*OsKAT1*, *OsSOS1* and *OsHKT1*) resulting in excessive Na^+^ and less K^+^ accumulation in shoots of *O. sativa* , inhibiting plant tolerance to salt stress (Fang et al. 2019; Zheng et al. 2024). Maintaining intracellular ion homeostasis is critical for plant tolerance to salt stress. Salt‐tolerant plants can prevent nutrient deficiency and cellular damage by sustaining higher K^+^ concentrations and limiting Na^+^ accumulation, thereby preserving a favourable K^+^/Na^+^ ratio under excessive salt conditions (Niu et al. 1995; Serrano et al. 1999; Yang and Guo 2018). In the present study, the changes of pectin contents in transgenic plants directly caused high K^+^ and low Na^+^ accumulations, leading to low Na^+^/K^+^ ratios in the leaves, stems, roots and their cell walls in *MsPG2*‐OE lines exposed to salt stress; but the contrast patterns of K^+^ and Na^+^ accumulations in tissues and cell walls were observed in *MsPG2*‐RNAi lines. The variation of Na^+^/K^+^ ratio in the tissues of transgenic plants is directly attributed to the changes of K^+^ and Na^+^ accumulations in the cell wall caused by pectin hydrolysis, rather than by ion homeostasis‐related genes *MsSOS1*, *MsNHX1* and *MsHKT1* (data not presented).

Stomatal closure is a rapid and essential adaptation response to salt and drought stresses, which functions to reduce water loss through transpiration and maintain cellular water homeostasis (Hedrich and Shabala 2018; Mittler and Blumwald 2015). In *A. thaliana* , *PMEI18* mutation increases pectin demethylesterification and reduces pectin degradation, leading to an increase in stomatal pore size, impairment of stomatal dynamics and increased plant sensitivity to drought stresses (Zhang, Guo, et al. 2023). Similarly, *OsPG1* mutants of *O. sativa* exhibit defective stomatal closure, leading to excessive water loss under hot conditions (Zou et al. 2024). The xylem in vascular tissues consists of interconnected vessel molecules that form long‐distance capillary channels for transporting water and minerals from the roots to the aerial parts (Lucas et al. 2013). The vessel surface is composed of proteins, lignin and pectins, whereas the proteins are mainly present in the intervessel pits and pectins are present at the outer edge of the pit membrane and in vessel‐parenchyma pits (Schenk et al. 2018). The gel property of pectins is one of the main determinants of vessel structure, and the cellulose microfibrils cross‐linked network structure affects cell wall extensibility (Mierczyńska et al. 2015). In the present study, overexpressing *MsPG2* reduced the stomatal aperture and vessel diameter of stems, which would control water loss from leaves, and reduce the transpiration pull and Na^+^ absorption and transportation from roots to shoots, consequently reducing Na^+^ accumulation in plants. On the whole, MsPG2 strongly influences the proportion of pectins in the cell wall composition, which affects cell wall extensibility, Na^+^/K^+^ ratio, stomatal aperture and vessel diameter and then affects alfalfa tolerance to salt stress.

### 3.2 MsNAC73 Directly Binds to the TC‐Rich Element of *MsPG2* Promoter and Negatively Regulates *MsPG2* Expression

Pectins are a polymer of heteropolysaccharides and play an important role in sustaining the rheological property and stability of the cell wall structure. Environmental stimuli and hormone levels can independently or synergistically affect the expression of *PG* genes and change pectin properties (Yang et al. 2018). ABA, ethylene and cold stress can upregulate the expressions of *MdPG1* and *SlPG* in apple ( *Malus domestica* ) and tomato ( *Solanum lycopersicum* ) (Sun et al. 2012; Tacken et al. 2010). Several transcription factors such as *Lateral Organ Boundaries Domain 18* (*LBD18*), auxin influx carrier LAX3, zinc‐finger protein AtC3H14 and ethylene response factor FcERF5 have been reported to regulate *PGs* expression in plants (Kim et al. 2014; Lee et al. 2015; Swarup et al. 2008; Wang et al. 2023). In recent years, more evidence has revealed the role of NAC transcription factors in regulating *PG* genes. MdNAC1‐L directly binds to *MdPG1*, and NAC TF SlNOR‐like1 binds to *SlPG2a*, which upregulates their expressions, promotes cell wall degradation and regulates fruit ripening and softening in *M. domestica* and *S. lycopersicum* (Gao et al. 2018; Peng et al. 2024; Su et al. 2024). In the present study, MsNAC73 was found to directly bind to the TC‐rich repeats in the *MsPG2* promoter and negatively regulate its expression.

NAC transcription factors have been shown to function as either positive or negative regulators in plant responses to salinity stress (Lu et al. 2018; Pan et al. 2024; Wang et al. 2022, 2020). Overexpression of *OsNAC3* enhances salt tolerance, whereas overexpression of *OsNAC2* reduces salt tolerance in *O. sativa* (Mao et al. 2018; Zhang, Long, et al. 2021). *MsNAC73* is highly expressed in stems and petioles (Fan et al. 2024), similar to the tissue expressions of *MsPG2*. Salt treatment led to a substantial increase or decrease of Na^+^ content and Na^+^/K^+^ ratio in the cell wall and plants of *MsNAC73*‐OE and ‐RNAi lines, respectively, which was consistent with salt‐induced phenotypes and accumulations of Na^+^ and K^+^ in *MsPG2*‐RNAi and ‐OE transgenic plants, respectively, suggesting that *MsNAC*73 functions as a main transcriptional repressor of *MsPG2* and regulates plant response to salt stress by sustaining Na^+^/K^+^ homeostasis.

### 3.3 The Interaction Between MsMPK3 and MsNAC73 Regulates Shoot Branching and Salt Tolerance of Alfalfa Under Normal and Salt‐Stressed Conditions Respectively

Phosphorylation is a critical posttranslational modification that modulates protein function and enables plants to respond to abiotic stresses rapidly. ZmNAC49 in *Z. mays* and IPA1 in *O. sativa* are phosphorylated by ZmMPK5 and OsMPK4, respectively, while AtCPK8 and AtMPK6 phosphorylate AtCAT3 and AtMYB15 in *A. thaliana* ; these phosphorylation events regulate plant responses to salt, drought and cold stresses (Jia et al. 2022; Kim et al. 2017; Xiang et al. 2021; Zou et al. 2015). In alfalfa, drought‐induced MsPIP2; 1 phosphorylation releases its interacted protein mMYB, and the mMYB translocates from membrane to nuclear, thereby regulating the balance between growth and drought responses of alfalfa (Lv et al. 2024). In the present study, MsNAC73 was found to interact with a mitogen‐activated protein kinase, MsMPK3, which is distributed in the nucleus, cytoplasm and cell membrane, and significantly upregulated under salt stress. The Thr‐123 was identified as an important phosphorylation site in MsNAC73. Western blot analysis revealed that salt stress increased the protein levels of MsMPK3, MsNAC73 and phosphorylation form MsNAC73^T123D^, but decreased the level of phosphorylation‐deficient form MsNAC73^T123A^ compared to the normal condition, indicating that salt stress induced MsNAC73 phosphorylation. The low levels of MsMPK3 and MsNAC73 under normal condition caused a weak interaction between MsMPK3 and MsNAC73, while the high levels of MsMPK3 and MsNAC73 under salinity condition strengthened their interaction and promoted MsNAC73 phosphorylation.

Protein phosphorylation modification is an important mechanism for modulating the balance between plant growth and stress response. The Na^+^/H^+^ antiporter SOS1 in *A. thaliana* is phosphorylated and activated by the kinase SOS2 under salt stress, promoting Na^+^ efflux to respond to salt stress. However, when salt stress is released, SOS1 is dephosphorylated by protein phosphatase 2C (PP2C), allowing the plant to transition back to normal growth (Fu et al. 2022). In salt‐stressed *O. sativa* , protein kinases (PK8, STRK1 and BAK1) activate CatC via phosphorylation, enhancing plant tolerance to salt and oxidative stresses. Upon removal of salt stress, phosphatase PC1 is activated and dephosphorylates CatC, which maintains appropriate H_2_O_2_ levels to sustain *O. sativa* growth and development (Liu et al. 2023). These findings suggest the importance of phosphorylation–dephosphorylation transitions in balancing plant growth and salt response. Our previous study demonstrated that MsNAC73 acts as a key transcriptional repressor of *MsPAE12*, which regulates alfalfa shoot branching via the IAA biosynthesis pathway under normal conditions (Fan et al. 2024). Thus, we speculated that the MsNAC73–MsMPK3 complex might coinfluence the shoot branching and salt stress response of alfalfa via phosphorylation–dephosphorylation of MsNAC73 to transcriptionally regulate the expressions of *MsPG2* and *MsPAE12* under normal and salt stress conditions. The transient expression assays in *N. benthamiana* and stable transformation assays in alfalfa hairy roots indicate that phosphorylation of MsNAC73 (MsNAC73^T123D^) attenuated its suppression of *MsPG2* expression, contributing to increased salt tolerance in *MsNAC73* ^*T123D*^‐OE hairy roots. While the dephosphorylation of MsNAC73 (MsNAC73^T123A^) promoted the expression of *MsPAE12*, leading to increased amounts of lateral roots and decreased root elongation in *MsNAC73* ^*T123A*^‐OE hairy roots under normal and salt stress conditions, respectively, which is consistent with the results of shoot branching increase in *MsNAC73*‐RNAi and *MsPAE12*‐OE lines (Fan et al. 2024). These results provide solid evidence that the MsNAC73–MsMPK3 complex synergistically regulates alfalfa shoot branching and salt response via activating *MsPAE12* and *MsPG2* expressions.

In conclusion, perennial herb plants have formed serial molecular adaptive mechanisms on the trade‐off between growth and survival to adapt to different environments. In the present study, a genetic network of MsNAC73‐MsMPK3–*MsPAE12*/*MsPG2* was identified and demonstrated to regulate alfalfa shoot branching and salt stress response. Salt stress induced MsMPK3 to phosphorylate MsNAC73, leading to upregulated *MsPG2* expression and increased alfalfa tolerance to salt stress via hydrolysing pectins, increasing cell wall extensibility, sustaining Na^+^/K^+^ homeostasis and reducing stomatal aperture and vessel diameter. While salt stress was released under normal condition, MsNAC73 was dephosphorylated or diminished phosphorylation, which promoted *MsPAE12* upregulation and increased shoot branching via the IAA biosynthesis pathway (Figure 7i). The MsNAC73‐MsMPK3‐*MsPAE12*/*MsPG2* represents a novel and crucial module that plays a key role in orchestrating the trade‐off between the branching and survival of alfalfa in response to different environments, and *MsNAC73* is an important gene for breeding alfalfa varieties with high production and salt resistance.

## 4 Materials and Methods

### 4.1 Plant Materials and Growth Conditions

Two alfalfa cultivars, WL‐525HQ (WL525) and Gannong No. 3 (GN3), procured from Beijing Rytway Seed Co. Ltd. and Gansu Agricultural University, respectively, were utilised as experimental materials in this study.

Seed germination and seedling culture were conducted following the protocol previously reported (Su et al. 2022). Environmental parameters for plant cultivation were maintained at 25°C with 70% relative humidity under controlled photoperiodic conditions (16 h/8 h light/dark period) with a light intensity of 400 μmol m^−2^ s^−1^. After 2 weeks, the tissues, including root tips, basal roots, buds, nodes, young leaves, mature leaves, old leaves, stems, petioles and stipules, were collected for RNA extraction.

NaCl treatment was applied to seed‐grown seedlings according to our previous report (Su et al. 2025). The 2‐week‐old seedlings were grown in 1/2 Hoagland nutrient solution (pH 5.8) with 100 mM NaCl. The leaves and roots were collected at 0, 1, 3, 6, 9, 12 and 24 h after treatment (0 h was a nontreated control). All harvested tissues were immediately cryopreserved at −80°C for subsequent analyses. For NaCl treatment on wild‐type and transgenic alfalfa, each line was grown in vermiculite irrigated with 1/2 Hoagland nutrient solution (pH 5.8) for 3 weeks, then treated with 0 or 400 mM NaCl for 21 days, and the phenotypes were recorded. The shoots and roots were harvested for physiological parameter assessment and RNA extraction. For NaCl treatment on transgenic alfalfa hairy roots, each line was treated with 0 or 100 mM NaCl for 7 days, and the phenotypes were recorded. The roots were collected for root length measurement and RNA extraction.

### 4.2 RNA Extraction, Expression Profiling and Sequence Analysis

Total RNA extraction was performed following the protocol described in a previous study (Wen et al. 2024). The expression levels of target genes were detected via real‐time RT‐qPCR utilising the Bio‐Rad CFX Duet detection system (Bio‐Rad, Hercules, CA, USA) in conjunction with Top Green qPCR SuperMix (TransGen Biotech, Beijing, China). The alfalfa elongation factor gene 1‐alpha (*MsEF‐α*) and actin 2 (*MsActin2*) were used as the internal standards. Primer sequences for RT‐qPCR are enumerated in Table S5. The experiments were conducted with a minimum of three independent biological replicates.

The conserved domain was predicted using the NCBI CD search tool (https://www.ncbi.nlm.nih.gov/Structure/cdd/wrpsb.cgi). The amino acid sequences of polygalacturonase (PG) genes from *A. thaliana* , *M. truncatula* and *M.* *sativa* were retrieved from the NCBI GenBank database (http://www.ncbi.nlm.nih.gov/genbank/). Multiple sequence alignment of the amino acid sequences was conducted using DNAMAN 8.0 software, and the phylogenetic tree was constructed using the Neighbour‐joining method by MEGA X (Tamura et al. 2013).

### 4.3 Plasmid Construction and Plant Transformation

For the generation of overexpression constructs, the complete coding sequence of *MsPG2* or *MsNAC73* was integrated into the pHB‐Flag expression vector. The RNA interference construct targeting *MsPG2* or *MsNAC73* was generated using the pHellsgate12 binary vector system. The obtained vectors were subsequently transformed into *A. tumefaciens* strain GV3101 or *Agrobacterium* *rhizogenes* LBA9402, and *Agrobacterium*‐mediated transgenic alfalfa and alfalfa hairy roots were generated as described previously (Li et al. 2020; Su et al. 2022).

The transgenic alfalfa plants (overexpressing *MsPG2* and RNAi‐induced knockdown of the *MsPG2* and *MsNAC73*), and the transformed alfalfa hairy roots (overexpressing *MsNAC73*, *MsNAC73* ^*T123A*^ and *MsNAC73* ^*T123D*^) were established from *M. sativa* cultivars GN3 and WL525 respectively. The *MsNAC73*‐overexpressing alfalfa plants were generated in our previous study (Fan et al. 2024).

### 4.4 Physiological Indices and Phenotype Analysis of Transgenic Plants

The malondialdehyde (MDA) content and hydrogen peroxide (H_2_O_2_) content were detected using reagent kits (Cominbio, Suzhou, China). Briefly, for MDA content determination, 0.1 g of fresh alfalfa sample was weighed, the instructions were followed to measure the absorbance at 532 and 600 nm, and the MDA contents were calculated by dry weight. For H_2_O_2_ content determination, 0.1 g of fresh alfalfa sample was weighed, the protocols were followed to measure the absorbance at 415 nm, and H_2_O_2_ contents were calculated by dry weight. The root elongation, electrolyte leakage (EL) and total chlorophyll content were measured following the method described by Lv et al. (2024) and Su et al. (2022) respectively. The content of K^+^ and Na^+^ in leaves, stems and roots was quantified according to the method described by Sun et al. (2025). Briefly, alfalfa leaf, stem and root samples were rinsed twice with distilled and deionised water, then wrapped in filter paper and placed in an oven at 60°C for 48 h. 0.02 g of the dry samples was weighed and transferred into 50‐mL centrifuge tubes. The samples were then digested using 1 mL of HNO_3_ and 1 mL of H_2_O_2_ in a digester set to 120°C for about 2 h. Following digestion, the samples were allowed to cool to room temperature and diluted to the same volume with deionised water. After standing for 24 h, 2 mL of the solution was aspirated, filtered and the Na^+^ and K^+^ contents were determined using an inductively coupled plasma emission spectrometer (Optima 800 ICP‐OES; PerkinElmer, Diamond, USA). Cell walls were extracted from leaves, stems and roots, and pectin and ion contents in the cell walls were measured following our previously described methodologies (Fan et al. 2022; Wang et al. 2017). The hemicellulose extraction method referred to Zhu et al. (2014).

The stomatal morphology was photographed using the VHX‐7000 digital microscope (Keyence, Japan), and aperture measurements were quantified using ImageJ analysis software. Images of 15 individual stomata from three biological replicates of each genotype were measured. The fifth internode of stems was cut by freehand sectioning and stained with phloroglucinol (Guo et al. 2023). The xylem vessels were photographed using the Eclipse Ti2 inverted microscope (Nikon, Japan). The root tips (0–1 cm) were treated with 20 mM HEPES (4 × 15 min), followed by subjection through a graded ethanol series (30%, 50%, 70%, 90% and 100%; 15 min each) and dehydrated with EM CPD 300 (Leica, Germany). The microstructures of cell walls on the root tips' surface were observed by Raman image‐scanning electron microscopy (RISE‐MAGNA, Tescan, Czech).

### 4.5 Yeast One‐Hybrid (Y1H) Assay

The promoter sequence of the target gene was cloned from alfalfa genomic DNA, and the sequence was analysed by the PlantCARE database (Lescot et al. 2002). For the Y1H assay, the coding sequence of *MsNAC73* was inserted into the pB42AD vector to create effectors, the promoter sequences or three tandem copies of predicted binding motifs were inserted into the pLacZ vector to create reporters; the various combinations of recombinants were cotransformed into the yeast strain EGY48 respectively. The interactions were evaluated on SD/Gal/Raf/−Trp/‐Ura plates with X‐gal for 2 days at 30°C.

### 4.6 Electrophoretic Mobility Shift (EMSA) Assay

The protein purification was performed following the previous study (Lv et al. 2021). Briefly, the full‐length coding sequence of *MsNAC73* was inserted into the pCold‐TF vector (TaKaRa, Dalian, China) and expressed in Rosetta (DE3) cells by adding 0.2 mM IPTG for 12 h at 16°C; then, Ni‐NTA agarose was used for purification. The EMSA was conducted following the Chemiluminescent EMSA Kit (GS009; Beyotime, Shanghai, China) instructions. The biotin‐labelled or unlabelled probes for the EMSA assay were synthesised by BGI Tech (Beijing, China).

### 4.7 Dual‐Luciferase (Dual‐LUC)

For reporter plasmids, the promoter sequences of *MsPG2* and *MsPAE12* were cloned into the pGreenII 0800‐LUC vector respectively. The pHB‐Flag (empty vector), MsNAC73‐Flag, MsNAC73^T123A^‐Flag, MsNAC73^T123D^‐Flag and MsMPK3‐YFP were created as effector plasmids. The effect and reporter plasmids were cotransformed into *N. benthamiana* leaves and incubated for 48 h at room temperature (Shi et al. 2018). The firefly LUC and REN activities were evaluated following the instructions (E1910; Promega, USA).

### 4.8 Subcellular Localisation Assay

The 35S::MsPG2‐YFP or 35S::MsMPK3‐YFP fusion constructs were expressed in *N. benthamiana* or *A. cepa* epidermal cells to study the MsPG2 or MsMPK3 subcellular localisation. The plasma membrane marker (CD3‐1007; PM‐marker) was cotransformed with MsMPK3‐YFP into the epidermal cells as the cytomembrane marker. The constructs and the respective negative controls were cotransformed into *N. benthamiana* or *A. cepa* leaves via *Agrobacterium*‐mediated transformation respectively. After incubation for 48 h at 25°C under dark conditions, fluorescence signals were observed using confocal laser microscopy (Leica TCS SP5‐II; Nussloch, Germany) with excitation at 514 nm for YFP detection. Plasmolysis experiments were performed according to Fan et al. (2022).

### 4.9 Pull‐Down Assay

The protein expression, purification and concentration were performed as described by Su et al. (2025). Then, the total protein extraction from alfalfa seedlings was performed according to Lv et al. (2021). The concentrated proteins (His‐MsNAC73 and His‐TF control) were incubated separately with Ni‐NTA agarose at 4°C for 3 h, and then equivalent volumes of total alfalfa protein were added to the respective Ni‐NTA agarose and further incubated at 4°C for 3 h. The Ni‐NTA agarose‐bound proteins were eluted with 1 × PBS buffer containing 400 mM imidazole. The eluates were subjected to LC–MS/MS analysis by BGI Tech (Shenzhen, China) for protein identification. The GPS 6.0 (http://gps.biocuckoo.cn) was employed to predict potential phosphorylation sites within the MsNAC73 protein sequence.

### 4.10 LC–MS/MS Assay

For the phosphorylation assay, the recombined vectors MsNAC73‐YFP, MsMPK3‐Flag and pHB‐Flag were constructed. *A. tumefaciens* strain GV3101 containing the corresponding recombinants was then coinfiltrated into *N. benthamiana* leaves. After 48‐h incubation in the darkness, the infiltrated leaves were collected. The protein extraction was performed by Bio‐Tech Pack Scientific (Beijing, China). The LC–MS/MS assay was performed to identify the phosphorylation sites of MsNAC73 by the Mass Spectrum Laboratory of Bio‐Tech Pack Technology Company (Beijing, China). To construct the plasmids, the mutated sequences of MsNAC73 were generated by site‐directed mutagenesis.

### 4.11 Yeast Two‐Hybrid (Y2H) Assay

The complete coding sequence of *MsNAC73* was cloned into the pGBKT7 vector, and the full‐length CDS of *MsMPK3* was inserted into the pGADT7 vector. The recombinant plasmids were cotransformed into yeast Y2H Gold competent cells and grown on DDO (SD/−Trp/−Leu) plates. Protein–protein interactions were evaluated by plating on QDO (SD/−Trp/−Leu/‐His/−Ade) + X‐*α*‐Gal + AbA medium.

### 4.12 Coimmunoprecipitation (Co‐IP) Assay

The recombined vectors MsNAC73‐Flag, MsNAC73^T123A^‐Flag, MsNAC73^T123D^‐Flag and MsMPK3‐YFP were constructed. *A. tumefaciens* strain GV3101 containing the corresponding recombinants was then coinfiltrated into *N. benthamiana* leaves. After 48‐h incubation in the darkness, the infiltrated leaves were collected and ground in liquid nitrogen. Total proteins were extracted using IP buffer (AWB‐0105; Abmart, Shanghai, China), and Co‐IP was performed using Anti‐GFP Nanobody Agarose Beads (KTSM1301; AlpalifeBio, Shenzhen, China) and Anti‐Flag Nanobody Agarose Beads (KTSM1360; AlpalifeBio, Shenzhen, China). For western blotting, commercial antibodies against Flag (Cell Signalling Technology, Shanghai, China), GFP (TransGen Biotech, Beijing, China), Phospho‐Thr (Cell Signalling Technology, Shanghai, China) and *β*‐actin (Abmart, Shanghai, China) were used.

### 4.13 Split Luciferase Complementation Assay

Two vectors, pCAMBIA1300‐nLUC (N terminus of LUC, nLUC) and pCAMBIA1300‐cLUC (C terminus of LUC, cLUC) were used for the split LUC complementation assay. *A. tumefaciens* GV3101 harbouring the MsMPK3‐nLUC, MsNAC73‐cLUC, MsNAC73^T123A^‐cLUC and MsNAC73^T123D^‐cLUC were coinfiltrated into *N. benthamiana* leaves based on experimental design. After 48‐h incubation in the darkness, the leaves were infiltrated with 0.5 mM D‐luciferin (Sagnon, Shanghai, China) and kept in the dark for 2 min. The images were captured by the Tanon5200 imaging system (Tanon, Shanghai, China).

### 4.14 Bimolecular Fluorescence Complementation (BiFC) Assay

The BiFC assay was employed to investigate the interaction of MsNAC73‐MsMPK3, MsNAC73^T123A^‐MsMPK3 and MsNAC73^T123D^‐MsMPK3 in planta. The vectors utilised here were pXY106 (N terminus of YFP, nYFP) and pXY104 (C terminus of YFP, cYFP) driven by the CaMV35S promoter. The constructs and the respective negative controls were cotransformed into *N. benthamiana* leaves via *Agrobacterium*‐mediated transformation respectively. After incubation for 48 h at 25°C under dark conditions, fluorescence signals were observed using confocal laser microscopy (Leica TCS SP5‐II, Nussloch, Germany) with excitation at 514 nm for YFP detection.

### 4.15 Statistical Analysis

All experiments were repeated at least three times independently. Data are means ± SE (*n* ≥ 3). Statistical significance was determined by ANOVA (Fisher LSD test) analysis using SAS at *p* < 0.05.

## Author Contributions

X.Y., Z.W. and Y.A. conceived and designed the research; X.Y. performed most of the experiments; N.F., L. Su, and Y.Z. provided assistance for alfalfa transformation; X.Y., L. Sun, L. Su, W.W., A.L., X.D., L.G., F.S. and P.Z. analysed the data; X.Y. wrote the manuscript; Y.A. critically revised the manuscript. All authors read and approved the manuscript.

## Conflicts of Interest

The authors declare no conflicts of interest.

## Supporting information


**Figure S1:** Identification of *MsNAC73*‐RNAi lines.
**Figure S2:** The physiological indices of wild‐type, *MsNAC73*‐OE and *MsNAC73*‐RNAi lines treated with or without salt.
**Figure S3:** Effects of MsNAC73 on the K^+^ content and Na^+^/K^+^ ratio in transgenic alfalfa.
**Figure S4:** The relative expression of *MsPAE12* in *MsNAC73*‐OE and *MsNAC73*–RNAi lines.
**Figure S5:** Sequence and phylogenetic tree analysis of MsPG2.
**Figure S6:** Expression pattern and subcellular localisation of MsPG2.
**Figure S7:** Identification of *MsPG2*‐OE and *MsPG2*‐RNAi lines.
**Figure S8:** The physiological indices of wild‐type, *MsPG2*‐OE and *MsPG2*‐RNAi lines treated with or without salt.
**Figure S9:** Effects of MsPG2 on the K^+^ content and Na^+^/K^+^ ratio in transgenic alfalfa.
**Figure S10:** Effects of MsNAC73 on pectin content in the cell wall of transgenic alfalfa.
**Figure S11:** Effects of MsPG2 on hemicellulose 1 content in the cell wall of transgenic alfalfa.
**Figure S12:** Effects of MsPG2 on the K^+^ content, Na^+^ content and Na^+^/K^+^ ratio in the cell wall of transgenic alfalfa.
**Figure S13:** Screening for potential proteins interacting with MsNAC73.
**Figure S14:** Potential phosphorylation sites of MsNAC73 analysed by LC–MS/MS.
**Figure S15:** Identification of transgenic alfalfa hairy roots.
**Figure S16:** The number of lateral roots in transgenic alfalfa hairy roots.
**Table S1:** The potential target genes of MsNAC73 selected from the MODMS database.
**Table S2:** The potential proteins interacted with MsNAC73.
**Table S3:** Prediction of potential phosphorylation sites in MsNAC73 by GPS (http://gps.biocuckoo.cn).
**Table S4:** Potential phosphorylation sites of MsNAC73 analysed by LC–MS/MS.
**Table S5:** Primers used in this study.

## Data Availability

All data are presented as figures. The data that support the findings of this study are available in the Supporting Information.
