# Supplementary material for: The MsNAC73–MsMPK3 Complex Modulates Salt Tolerance and Shoot Branching of Alfalfa via Activating MsPG2 and MsPAE12 Expressions
Source: Plant Biotechnol J. 2025 Aug 20;23(12):5635–53. doi: 10.1111/pbi.70323 (PMC12665078; doi:10.1111/pbi.70323)
Supplement: Supplementary file 1 — Figure S1: Identification of MsNAC73‐RNAi lines. Figure S2: The physiological indices of wild‐type, MsNAC73‐OE and MsNAC73‐RNAi lines treated with or without salt. Figure S3: Effects of MsNAC73 on the K+ content and Na+/K+ ratio in transgenic alfalfa. Figure S4: The relative expression of MsPAE12 in MsNAC73‐OE and MsNAC73–RNAi lines. Figure S5: Sequence and phylogenetic tree analysis of MsPG2. Figure S6: Expression pattern and subcellular localisation of MsPG2. Figure S7: Identification of MsPG2‐OE and MsPG2‐RNAi lines. Figure S8: The physiological indices of wild‐type, MsPG2‐OE and MsPG2‐RNAi lines treated with or without salt. Figure S9: Effects of MsPG2 on the K+ content and Na+/K+ ratio in transgenic alfalfa. Figure S10: Effects of MsNAC73 on pectin content in the cell wall of transgenic alfalfa. Figure S11: Effects of MsPG2 on hemicellulose 1 content in the cell wall of transgenic alfalfa. Figure S12: Effects of MsPG2 on the K+ content, Na+ content and Na+/K+ ratio in the cell wall of transgenic alfalfa. Figure S13: Screening for potential proteins interacting with MsNAC73. Figure S14: Potential phosphorylation sites of MsNAC73 analysed by LC–MS/MS. Figure S15: Identification of transgenic alfalfa hairy roots. Figure S16: The number of lateral roots in transgenic alfalfa hairy roots. Table S1: The potential target genes of MsNAC73 selected from the MODMS database. Table S2: The potential proteins interacted with MsNAC73. Table S3: Prediction of potential phosphorylation sites in MsNAC73 by GPS (http://gps.biocuckoo.cn). Table S4: Potential phosphorylation sites of MsNAC73 analysed by LC–MS/MS. Table S5: Primers used in this study. [file PBI-23-5635-s001.docx]

**The MsNAC73-MsMPK3 complex modulates ﻿salt tolerance and shoot branching of alfalfa via activating *MsPG2* and *MsPAE12* expressions**

Xiangkai You^1, 2 †^, ﻿Nana Fan^3 †^, Yuehua Zhang^4, 5 †^, Linjie Sun^1^, Liantai Su^6^, Wuwu Wen^1^, ﻿Aimin Lv^7^, Xinyu Dai^4^ , Li Gao^1^, Fengling Shi^5^, Peng Zhou^1^, Zhaoming Wang^4＊^, Yuan An^1, 8＊^

**The following Supporting Information is available for this article:**

**Figure S1** **Identification of *MsNAC73*-RNAi lines.**

(a) PCR detection for the *kanamycin* resistance gene in *MsNAC73*-RNAi lines. M: *Trans*2K^®^ Plus DNA marker. +: Positive control. -: Negative control. 1-5: Samples. (b) Relative expressions of *MsNAC73* in wild-type (WT) and *MsNAC73*-RNAi lines. GN3 represents the WT of alfalfa; Ri7B and Ri10B represent two independent *MsNAC73*-RNAi lines. *MsEF-α* and *MsActin2* were used as the internal standards. Data are means ± SE (n ≥ 3). Bars with different letters indicate significant differences at *P* < 0.05.


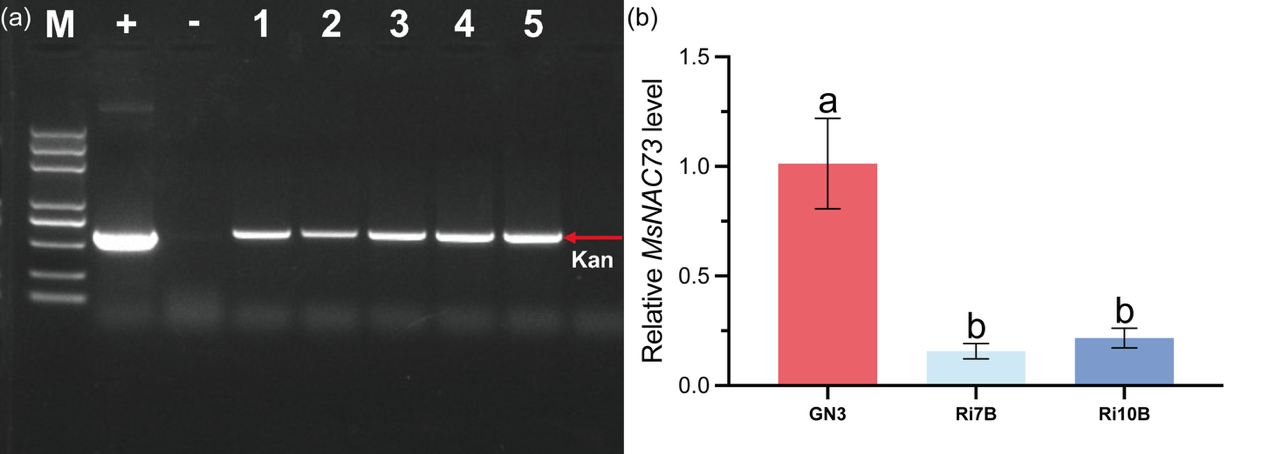


**Figure S2 The physiological indices of wild-type,** ***MsNAC73*-OE, and *MsNAC73*-RNAi lines treated with or without salt.**

(a) The wild-type (WT) and transgenic alfalfa were grown in vermiculite irrigated with ^1^/_2_ Hoagland nutrient solution (pH 5.8), then treated with 0 mM or 400 mM NaCl for 21 d**.** The total chlorophyll content of alfalfa treated with 0 or 400 mM NaCl was measured. (b) The H_2_O_2_ content of alfalfa treated with 0 or 400 mM NaCl was measured. GN3 represents the WT of alfalfa; OE11 and OE13 represent two independent *MsNAC73*-OE lines; Ri7B and Ri10B represent two independent *MsNAC73*-RNAi lines. FW: fresh weight; DW: dry weight. Data are means ± SE (n ≥ 3). Bars with different letters indicate significant differences at *P* < 0.05.

**
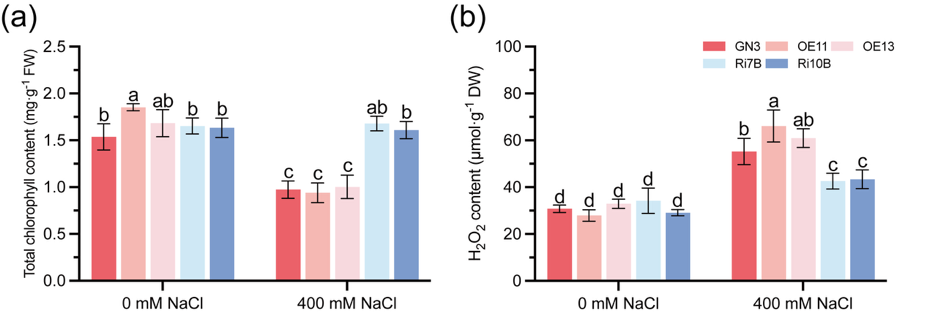
**

**Figure S3** **Effects of MsNAC73 on the K⁺ content and Na^+^/K^+^ ratio in transgenic alfalfa.**

(a-c) The contents of K⁺ in leaves (a), stems (b), and roots (c) in transgenic alfalfa treated with 0 or 400 mM NaCl were measured. (d-f) The Na^+^/K^+^ ratio in leaves (d), stems (e), and roots (f) was calculated. GN3 represents the wild-type of alfalfa; OE11 and OE13 represent two independent *MsNAC73*-OE lines; Ri7B and Ri10B represent two independent *MsNAC73*-RNAi lines. DW: Dry weight. Data are means ± SE (n ≥ 3). Bars with different letters indicate significant differences at *P* < 0.05.


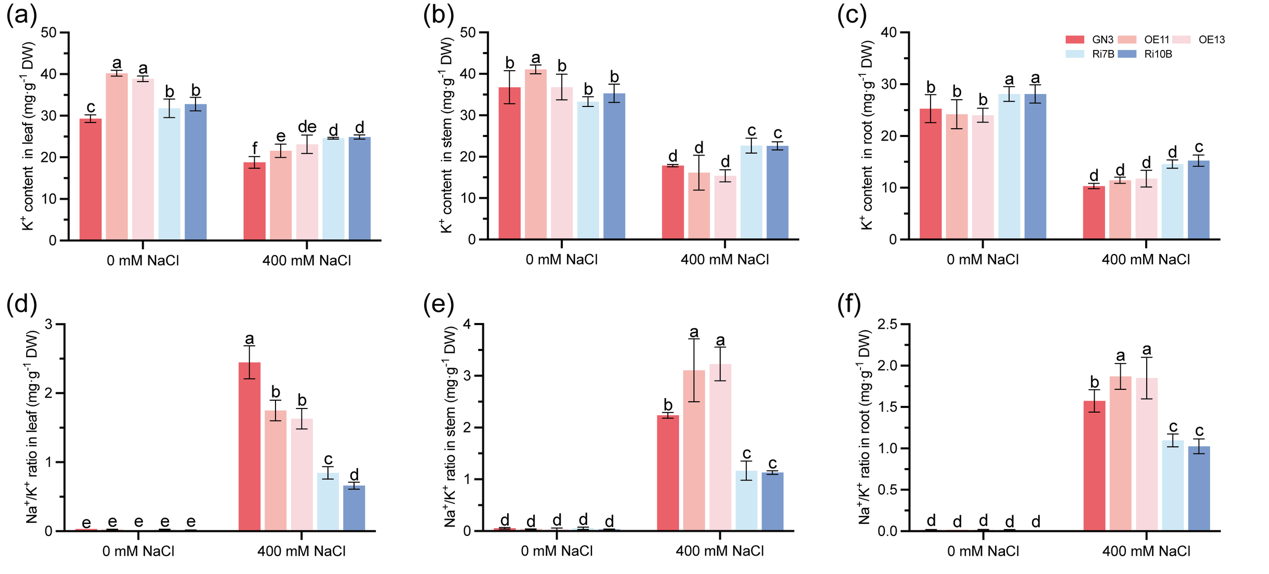


**Figure S4 The relative expression of *MsPAE12* in *MsNAC73*-OE and *MsNAC73*–RNAi lines.**

The relative expression of *MsPAE12* in wild-type (GN3), *MsNAC73*-OE lines (OE11, OE13), and *MsNAC73*-RNAi lines (Ri7B, Ri10B). *MsEF-α* and *MsActin2* were used as the internal standards. Data are means ± SE (n ≥ 3). Bars with different letters indicate significant differences at *P* < 0.05.

**
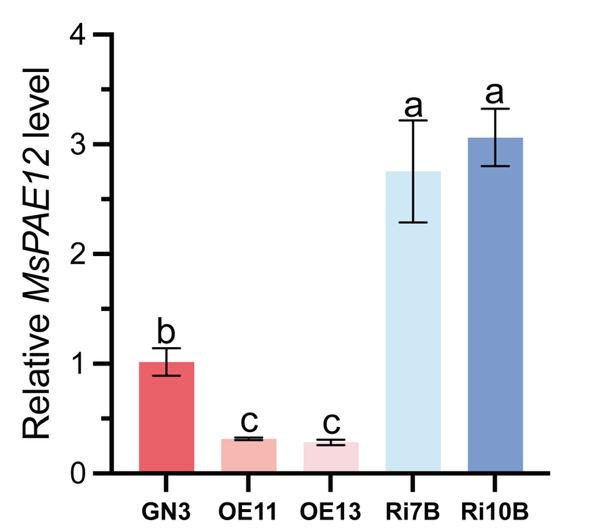
**

**Figure S5 Sequence and phylogenetic tree analysis of MsPG2.**

(a) The conserved domain analysis of MsPG2 by the NCBI CD search tool. (b) The alignment of the deduced amino acid sequence of MsPG2 with 1 PG from *M. truncatula* (AES59005.1). The red asterisk indicates that the MsPG2 contains four typical conserved residues of PG protein. (c) The phylogenetic tree was constructed using the Neighbor-joining method by MEGA X. Bootstrap values are shown as percentages (1000 replicates).


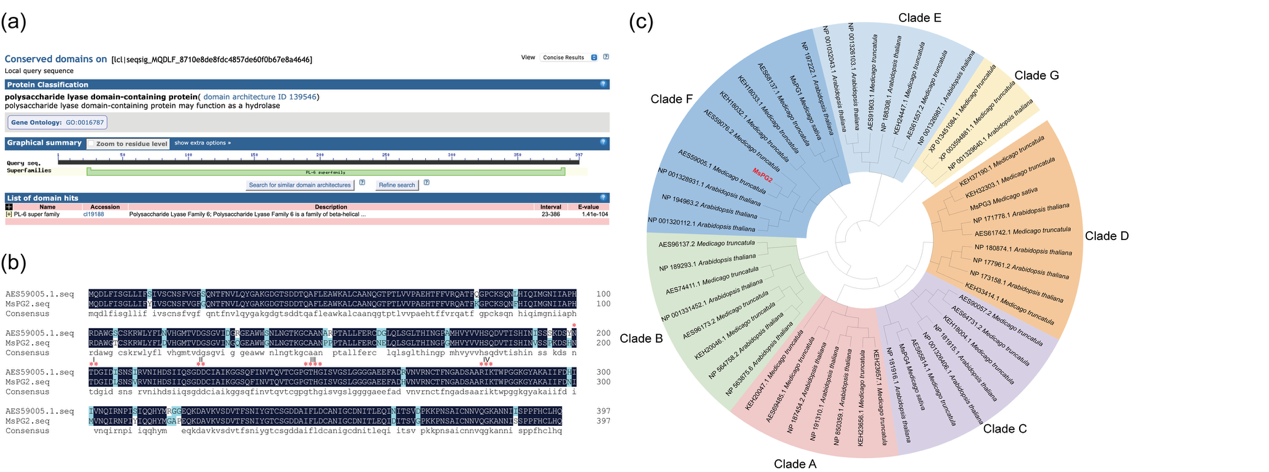


**Figure S6 Expression pattern and subcellular localization of MsPG2.**

(a) The relative expression of *MsPG2* in different tissues. *MsEF-α* and *MsActin2* were used as the internal standards. RT: root tip; BR: basal root; ﻿YL: young leaf; ML: mature leaf; OL: old leaf. (b) The subcellular localization of the 35S::MsPG2-YFP in *Nicotiana benthamiana* (upper six images) and *A. cepa* (lower six images). The fluorescent signals were observed using confocal microscopy. White and red arrowheads indicate the plasma membrane and cell wall, respectively. The 35S::YFP was used as a control. Bar = 50 μm. (c) The relative expression of *MsPG2* in leaves and roots of two-week-old alfalfa seedlings treated with 100 mM NaCl at different time points. ﻿RNA extracted from leaves and roots was used for RT-qPCR, *MsEF-α* and *MsActin2* were used as the internal standards. Data are means ± SE (n ≥ 3). Bars with different letters indicate significant differences at *P* < 0.05.


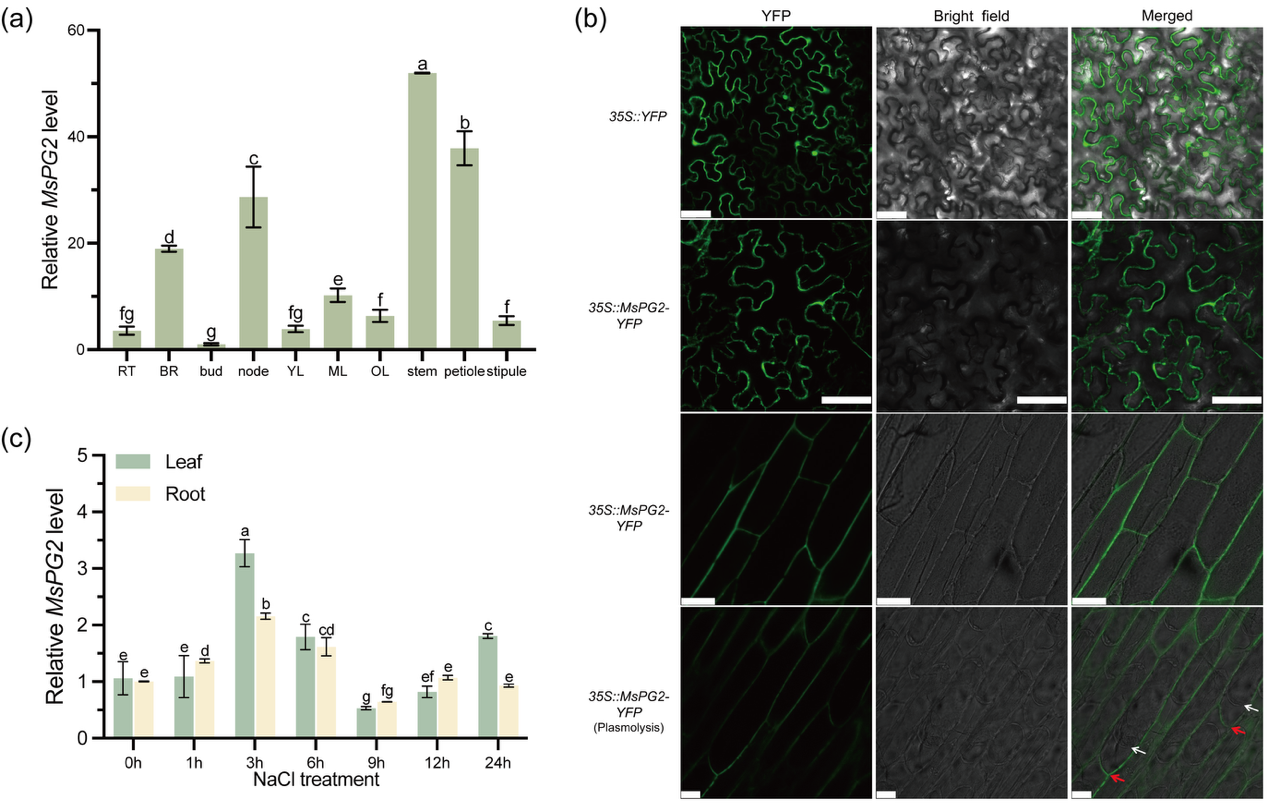


**Figure S7 Identification of *MsPG2*-OE and *MsPG2*-RNAi lines.**

(a) PCR detection for the *hygromycin* resistance gene in *MsPG2*-OE lines. M: *Trans*2K^®^ Plus DNA marker. +: Positive control. -: Negative control. 1-6: Samples. (b) PCR detection for the *kanamycin* resistance gene in *MsPG2*-RNAi lines. M: *Trans*2K^®^ Plus DNA marker. +: Positive control. -: Negative control. 1-6: Samples. (c-d) Relative expressions of *MsPG2* in wild-type (WT), *MsPG2*-OE lines (c), and *MsPG2*-RNAi lines (d). GN3 represents the WT of alfalfa, OE1 and OE19 represent two independent *MsPG2*-OE lines, and Ri5B and Ri6B represent two independent *MsPG2*-RNAi lines. *MsEF-α* and *MsActin2* were used as the internal standards. Data are means ± SE (n ≥ 3). Bars with different letters indicate significant differences at *P* < 0.05.

**
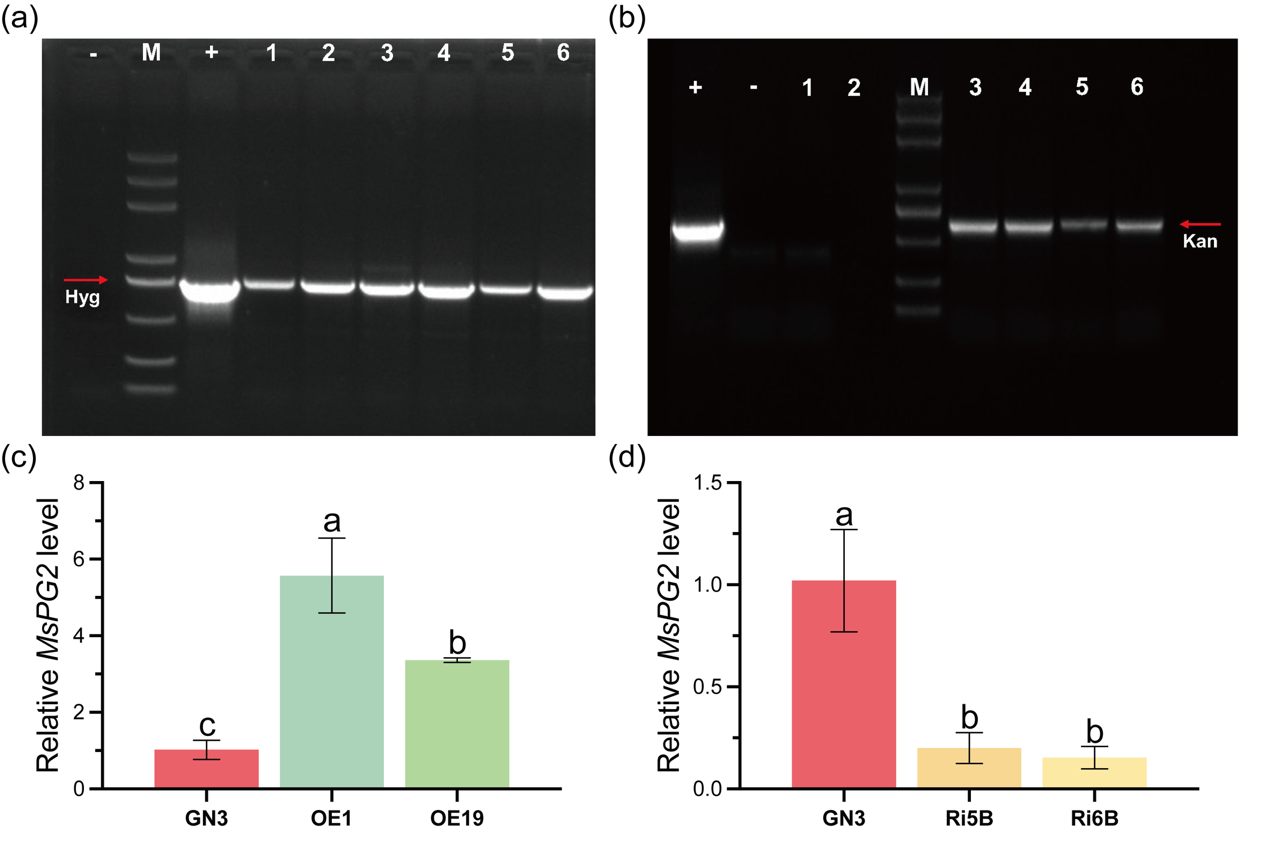
**

**Figure S8 The physiological indices of wild-type, *MsPG2*-OE, and *MsPG2*-RNAi lines treated with or without salt.**

(a-c) The wild-type (WT) and transgenic alfalfa were grown in vermiculite irrigated with ^1^/_2_ Hoagland nutrient solution (pH 5.8), then treated with 0 mM or 400 mM NaCl for 21 d**.** The total chlorophyll content (a), MDA content (b), and H_2_O_2_ content (c) of alfalfa treated with 0 or 400 mM NaCl were measured. GN3 represents the WT of alfalfa; OE1 and OE19 represent two independent *MsPG2*-OE lines; Ri5B and Ri6B represent two independent *MsPG2*-RNAi lines. FW: fresh weight; DW: dry weight. Data are means ± SE (n ≥ 3). Bars with different letters indicate significant differences at *P* < 0.05.

**
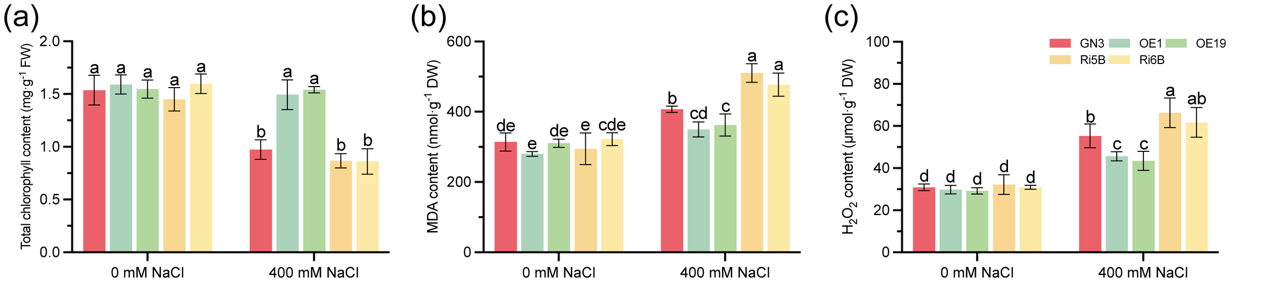
**

**Figure S9** **Effects of MsPG2 on the K⁺ content and Na^+^/K^+^ ratio in transgenic alfalfa.**

(a-c) The contents of K⁺ in leaves (a), stems (b), and roots (c) in transgenic alfalfa treated with 0 or 400 mM NaCl were measured. (d-f) The Na^+^/K^+^ ratio in leaves (d), stems (e), and roots (f) was calculated. GN3 represents the wild-type of alfalfa; OE1 and OE19 represent two independent *MsPG2*-OE lines; Ri5B and Ri6B represent two independent *MsPG2*-RNAi lines. DW: Dry weight. Data are means ± SE (n ≥ 3). Bars with different letters indicate significant differences at *P* < 0.05.

**
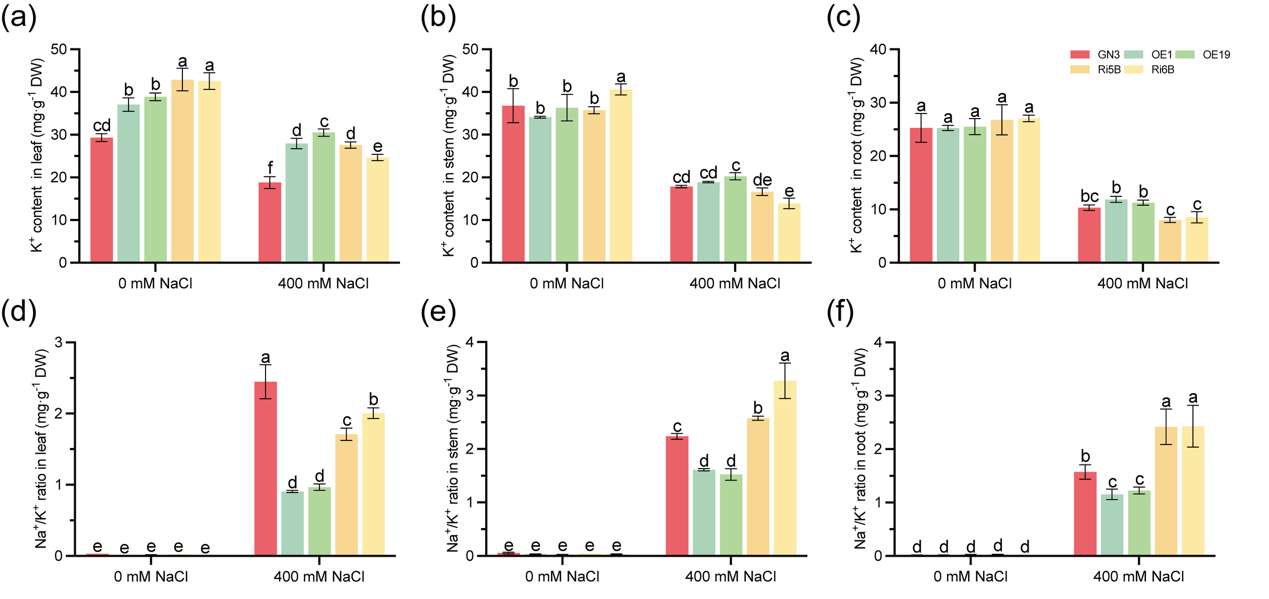
**

**Figure S10 Effects of MsNAC73 on pectin content in the cell wall of transgenic alfalfa.**

The uronic acid content in pectins extracted from leaves (a), stems (b), and roots (c) of alfalfa treated with 0 or 400 mM NaCl. GN3 represents the WT of alfalfa; OE11 and OE13 represent two independent *MsNAC73*-OE lines; Ri7B and Ri10B represent two independent *MsNAC73*-RNAi lines. CW: cell wall. Data are means ± SE (n ≥ 3). Bars with different letters indicate significant differences at *P* < 0.05.


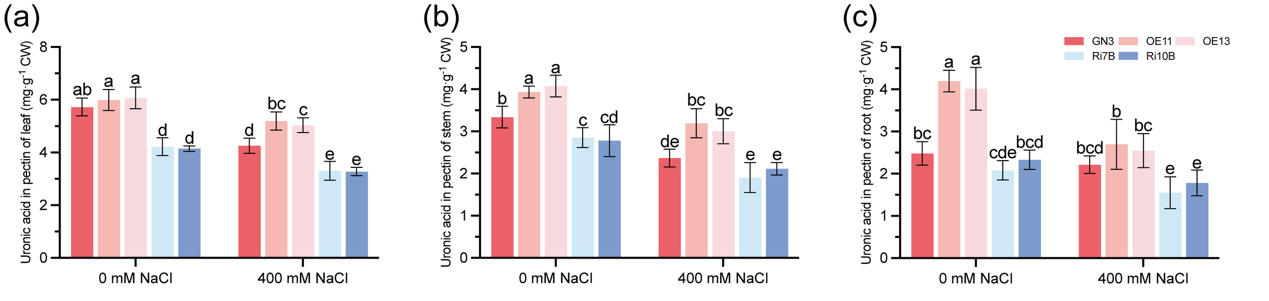


**Figure S11 Effects of MsPG2 on hemicellulose 1 content in the cell wall of transgenic alfalfa.**

The total sugar content in hemicellulose 1 extracted from leaves (a), stems (b), and roots (c) of alfalfa treated with 0 or 400 mM NaCl. GN3 represents the WT of alfalfa; OE1 and OE19 represent two independent *MsPG2*-OE lines; Ri5B and Ri6B represent two independent *MsPG2*-RNAi lines. CW: cell wall. Data are means ± SE (n ≥ 3). Bars with different letters indicate significant differences at *P* < 0.05.


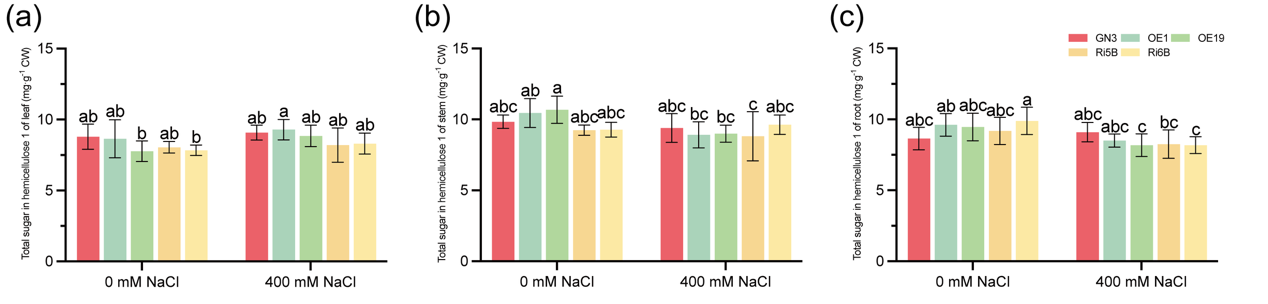


**Figure S12** **Effects of MsPG2 on the K⁺ content, Na^+^ content, and Na^+^/K^+^ ratio in the cell wall of transgenic alfalfa.**

(a-c) The contents of K⁺ in the cell wall of leaves (a), stems (b), and roots (c) in transgenic alfalfa treated with 0 or 400 mM NaCl. (d-f) The contents of Na⁺ in the cell walls of leaves (d), stems (e), and roots (f) in transgenic alfalfa treated with 0 or 400 mM NaCl. (g-i) The Na^+^/K^+^ ratio in the cell wall of leaves (g), stems (h), and roots (i) was calculated. GN3 represents the wild-type of alfalfa; OE1 and OE19 represent two independent *MsPG2*-OE lines; Ri5B and Ri6B represent two independent *MsPG2*-RNAi lines. CW: Cell wall. Data are means ± SE (n ≥ 3). Bars with different letters indicate significant differences at *P* < 0.05.


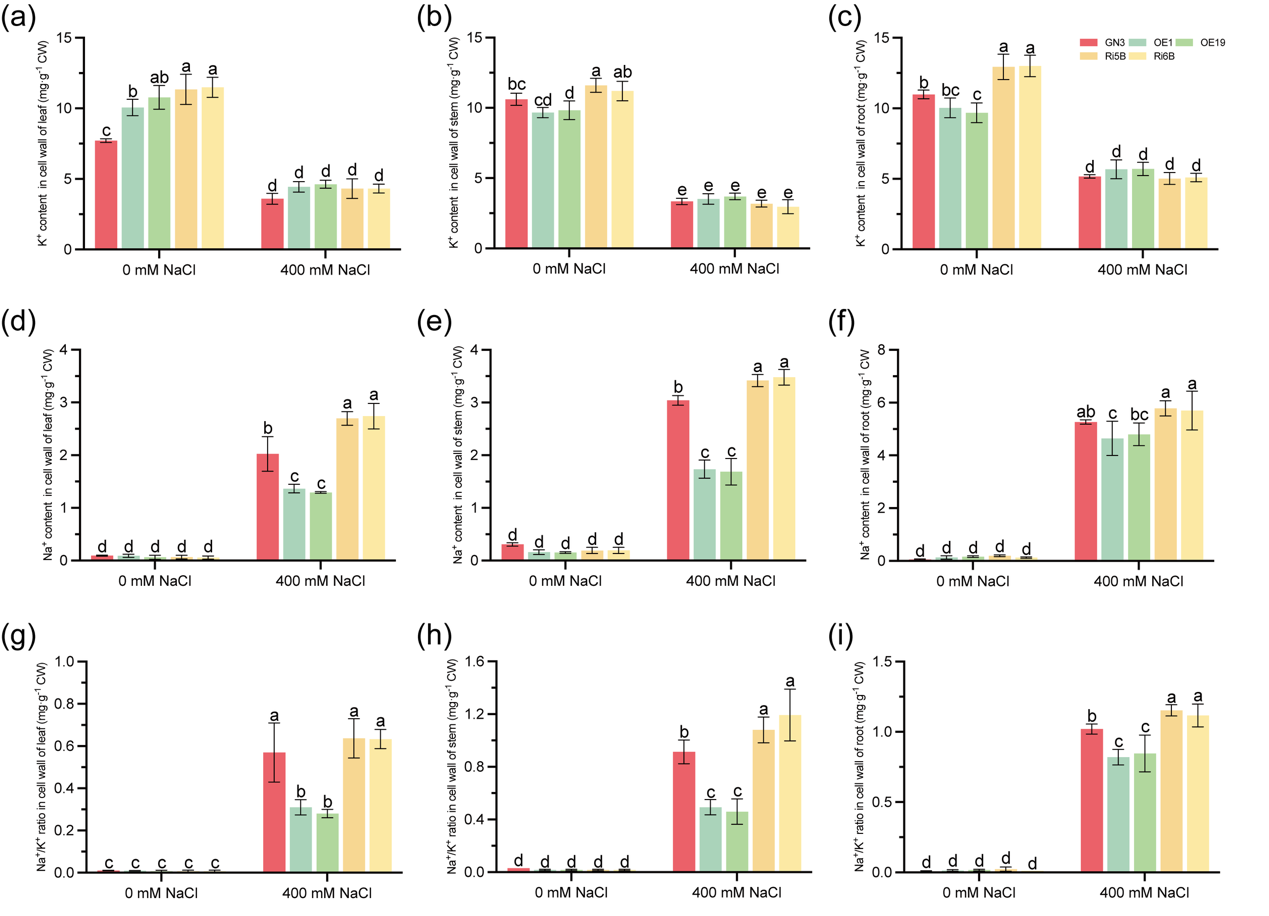


**Figure S13 Screening for potential proteins interacting with MsNAC73.**

Lanes 1 and 6 indicated effluent through Ni-NTA after alfalfa total protein incubated with pCold control. Lane 2 indicated the first elution of Ni-NTA with 400 mM imidazole after incubation of pCold control with alfalfa total protein, Lane 3 indicated the second elution of Ni-NTA with 400 mM imidazole after incubation of pCold control with alfalfa total protein, Lane 4 indicated the third elution of Ni-NTA with 400 mM imidazole after incubation of pCold control with alfalfa total protein, Lane 5 indicated the fourth elution of Ni-NTA with 400 mM imidazole after incubation of pCold control with alfalfa total protein. Lane 7 indicated the first elution of Ni-NTA with 400 mM imidazole after incubation of His-MsNAC73 with alfalfa total protein, Lane 8 indicated the second elution of Ni-NTA with 400 mM imidazole after incubation of His-MsNAC73 with alfalfa total protein, Lane9 indicated the third elution of Ni-NTA with 400 mM imidazole after incubation of His-MsNAC73 with alfalfa total protein, Lane 10 indicated the fourth elution of Ni-NTA with 400 mM imidazole after incubation of His-MsNAC73 with alfalfa total protein.

**
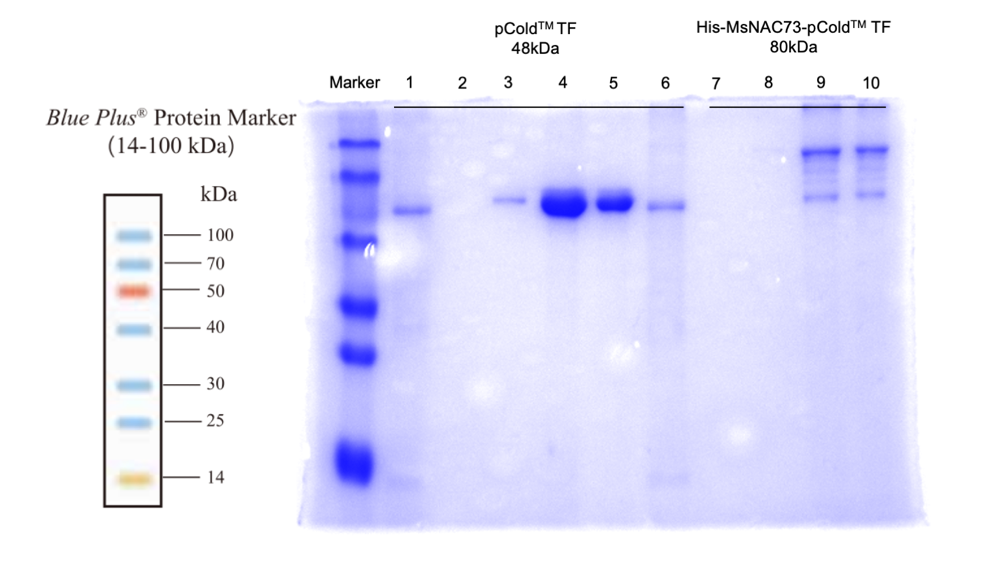
**

**Figure S14 Potential phosphorylation sites of MsNAC73 analyzed by LC-MS/MS.**

(a-b) The LC-MS/MS assay was performed to identify the phosphorylation sites of MsNAC73 by the Mass Spectrum Laboratory of Bio-Tech Pack Technology Company (Beijing, China).

**
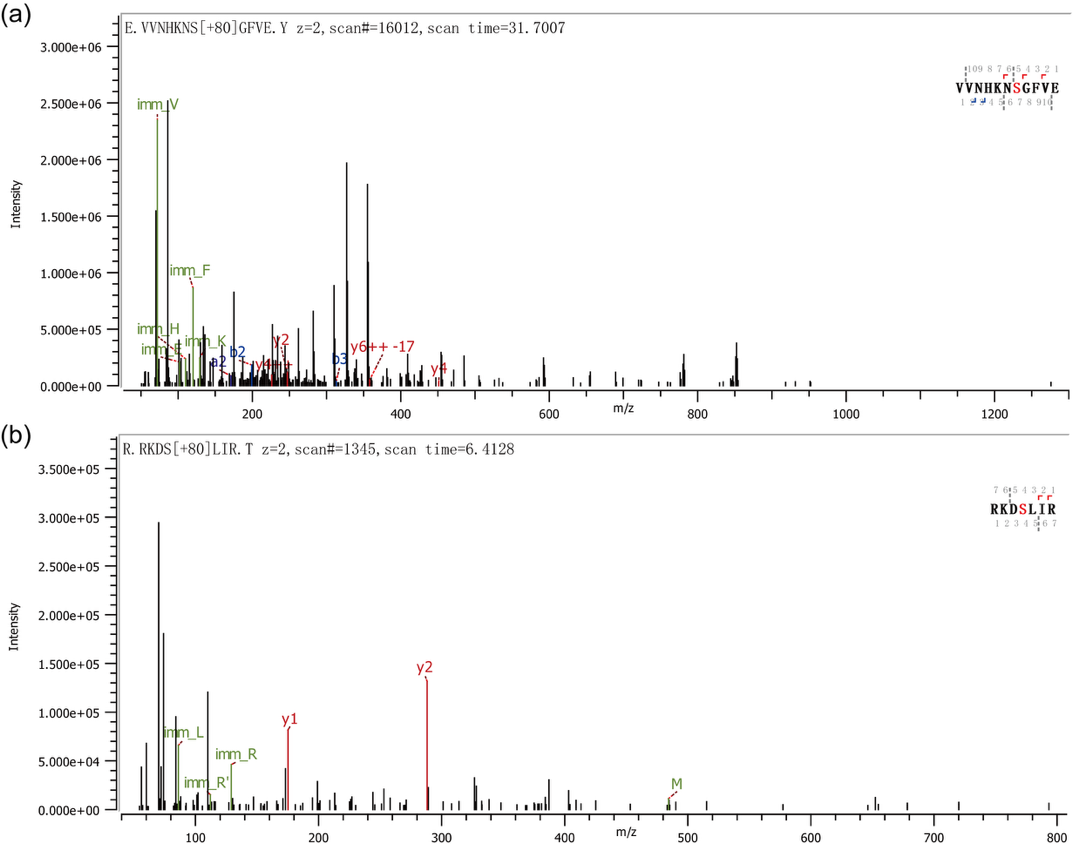
**

**Figure S15 Identification of transgenic alfalfa hairy roots.**

(a) PCR detection for the *hygromycin* resistance gene in *MsNAC73*-OE lines, *MsNAC73^T123A^*-OE lines, and *MsNAC73^T123D^*-OE lines. M: *Trans*2K^®^ Plus DNA marker. +: Positive control. -: Negative control. n1-n4: *MsNAC73*-OE lines samples. a1-a4: *MsNAC73^T123A^*-OE lines samples. d1-d4: *MsNAC73^T123D^*-OE lines samples. (b) Relative expression of *MsNAC73* in *MsNAC73*-OE lines. The vector represents the control line, and OEn1 and OEn4 represent two independent *MsNAC73*-OE lines. (c) Relative expression of *MsNAC73* in *MsNAC73^T123A^*-OE lines. The vector represents the control line, and OEa1 and OEa4 represent two independent *MsNAC73^T123A^*-OE lines. (d) Relative expression of *MsNAC73* in *MsNAC73^T123D^*-OE lines. The vector represents the control line, and OEd2 and OEd3 represent two independent *MsNAC73^T123D^*-OE lines. *MsEF-α* and *MsActin2* were used as the internal standards. Data are means ± SE (n ≥ 3). Bars with different letters indicate significant differences at *P* < 0.05.

**
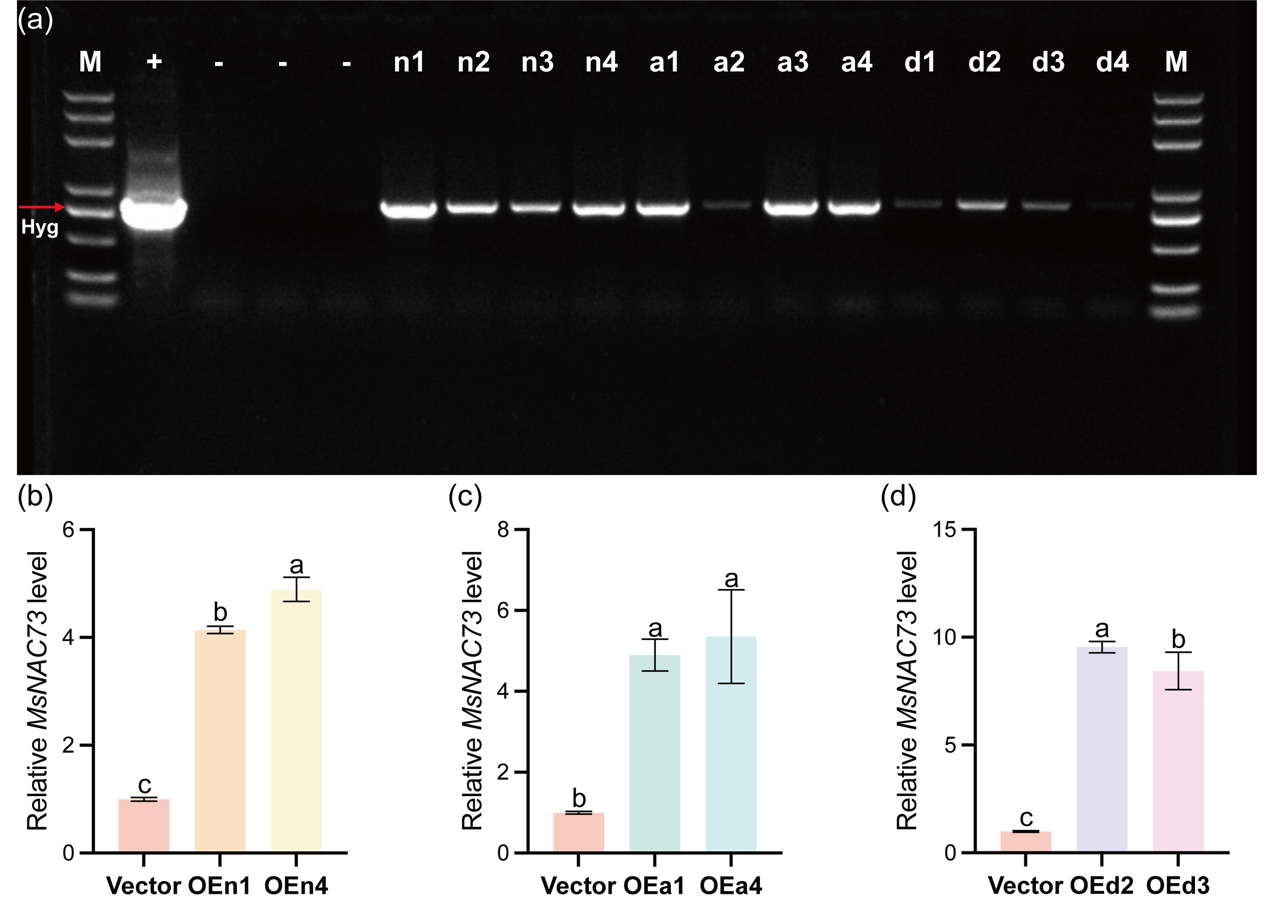
**

**Figure S16 The number of lateral roots ﻿in transgenic alfalfa hairy roots.**

The vector represents the control line; OEn1 and OEn4 represent two independent *MsNAC73*-OE lines; OEa1 and OEa4 represent two independent *MsNAC73^T123A^*-OE lines; OEd2 and OEd3 represent two independent *MsNAC73^T123D^*-OE lines. Data are means ± SE (n ≥ 3). Bars with different letters indicate significant differences at *P* < 0.05.


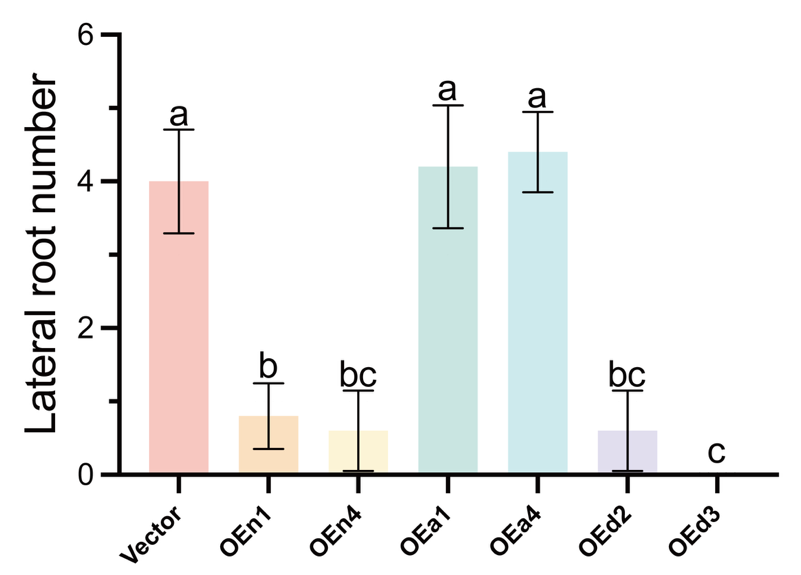


**Table S1 The potential target genes of MsNAC73 selected from the MODMS database.**

| Gene_ID | salt_0h | salt_3h | salt_6h | salt_12h | narr OG desc | PFAMs |
| --- | --- | --- | --- | --- | --- | --- |
| MsG0280010788.01.T01 (MsNAC73) | 3.27 | 4.13 | 4.74 | 3.9 | No apical meristem (NAM) protein | NAM |
| MsG0180000606.01.T01 (MsPG2) | 32.81 | 56.09 | 40.37 | 37.82 | Polygalacturonase | Glyco_hydro_28 |
| MsG0180000805.01.T01 | 74.91 | 46.06 | 70.12 | 57.85 | pectinesterase inhibitor | PMEI,Pectinesterase |
| MsG0180004763.01.T01 | 38.94 | 68.3 | 54.81 | 46.14 | Polygalacturonase | DNA_pol_A_exo1,Glyco_hydro_28,Pectate_lyase_3 |
| MsG0180005076.01.T01 | 133.23 | 102.27 | 59 | 180.91 | Polygalacturonase | Cornifin,LRRNT_2,LRR_1,LRR_4,LRR_6,LRR_8 |
| MsG0280008106.01.T01 | 0.05 | 0.37 | 0.11 | 0.32 | Polygalacturonase QRT3-like | Beta_helix,Pectate_lyase_3 |
| MsG0280011393.01.T01 | 29.97 | 84.03 | 45.71 | 79.58 | Polygalacturonase | Glyco_hydro_28 |
| MsG0480019217.01.T01 | 242.38 | 186.53 | 131.1 | 180.98 | Acts in the modification of cell walls via demethylesterification of cell wall pectin | PMEI,Pectinesterase |
| MsG0480023454.01.T01 | 73.48 | 81.01 | 83.95 | 74.43 | pectinesterase inhibitor | PMEI,Pectinesterase |
| MsG0480023798.01.T01 | 48.67 | 131.82 | 68.17 | 62.72 | pectinesterase | Pectinesterase |
| MsG0780036901.01.T01 | 185.96 | 546.9 | 265.84 | 493.93 | Polygalacturonase | Cornifin,LRRNT_2,LRR_1,LRR_4,LRR_6,LRR_8 |
| MsG0780038305.01.T01 | 299.34 | 731.58 | 354.31 | 476.82 | Acts in the modification of cell walls via demethylesterification of cell wall pectin | PMEI,Pectinesterase |
| MsG0780038306.01.T01 | 225.22 | 355.26 | 193.18 | 249.21 | Acts in the modification of cell walls via demethylesterification of cell wall pectin | PMEI,Pectinesterase |
| MsG0780038307.01.T01 | 413.03 | 173.89 | 301.67 | 79.61 | Acts in the modification of cell walls via demethylesterification of cell wall pectin | PMEI,Pectinesterase |
| MsG0780041316.01.T01 | 46.75 | 77.66 | 56.21 | 53.11 | pectinesterase inhibitor | DUF538,PMEI,Pectinesterase |
| MsG0880041983.01.T01 | 0.34 | 0.25 | 0.21 | 0.12 | Polygalacturonase | Glyco_hydro_28 |
| MsG0880042039.01.T01 | 0.16 | 0.13 | 0.1 | 0.06 | Polygalacturonase | Glyco_hydro_28 |
| MsG0880042835.01.T01 | 102.14 | 208.54 | 174.98 | 122.41 | pectinesterase | DUF677,PMEI,Pectinesterase |
| MsG0880044656.01.T02 | 63.51 | 285.06 | 90.73 | 219.82 | Polygalacturonase | Cornifin,LRRNT_2,LRR_1,LRR_4,LRR_6,LRR_8 |

**Table S2 The potential proteins interacted with MsNAC73.**

| ﻿NO. | Protein_ID | Description |
| --- | --- | --- |
| 1 | tr\|A0A4P8F0D4\|A0A4P8F0D4_MEDSA | ATP synthase subunit beta |
| 2 | tr\|A0A1B3T043\|A0A1B3T043_MEDSA | Ribulose bisphosphate carboxylase large chain |
| 3 | tr\|Q8GTY4\|Q8GTY4_MEDSA | Ribulose bisphosphate carboxylase/oxygenase activase, chloroplastic (Fragment) |
| 4 | tr\|C8CGU0\|C8CGU0_MEDSA | Glyceraldehyde-3-phosphate dehydrogenase |
| 5 | tr\|G4XKY0\|G4XKY0_9CARY | Vacuolar proton pump subunit B |
| 6 | tr\|Q9SBR8\|Q9SBR8_MEDSV | L3 Ribosomal protein |
| 7 | sp\|A4ULF8\|METK_MEDSF | S-adenosylmethionine synthase |
| 8 | tr\|G7JAX4\|G7JAX4_MEDTR | Actin 2 |
| 9 | tr\|Q949G6\|Q949G6_MEDSF | Tubulin beta chain |
| 10 | tr\|Q93WS1\|Q93WS1_MEDSA | Selenium binding protein |
| 11 | tr\|Q5MGA8\|Q5MGA8_MEDSA | Heat shock protein 70 |
| 12 | sp\|O24076\|GBLP_MEDSA | Guanine nucleotide-binding protein subunit beta-like protein |
| 13 | tr\|A0A221C697\|A0A221C697_MEDSF | Elongation factor 1-alpha |
| 14 | tr\|Q9SDP6\|Q9SDP6_MEDSA | phosphoenolpyruvate carboxykinase (ATP) (Fragment) |
| 15 | tr\|O24483\|O24483_MEDSA | Asparagine synthetase [glutamine-hydrolyzing] |
| 16 | tr\|O81391\|O81391_MEDSA | Chlorophyll a-b binding protein, chloroplastic |
| 17 | tr\|A0A4Y5P838\|A0A4Y5P838_MEDSF | Translation elongation factor-2 |
| 18 | sp\|Q9BAE0\|FTSH_MEDSA | ATP-dependent zinc metalloprotease FTSH, chloroplastic |
| 19 | tr\|A0A6B7JET2\|A0A6B7JET2_MEDSF | NAC domain-containing protein NAC44 |
| 20 | tr\|Q9ZP90\|Q9ZP90_MEDSA | Ferritin |
| 21 | sp\|O65194\|RBS_MEDSA | Ribulose bisphosphate carboxylase small subunit, chloroplastic |
| 22 | tr\|A0A4P8F1K7\|A0A4P8F1K7_MEDSF | ATP synthase subunit alpha |
| 23 | tr\|Q40367\|Q40367_MEDSA | Peroxidase (Fragment) |
| 24 | tr\|E5DKC8\|E5DKC8_MEDSA | ATP synthase subunit alpha (Fragment) |
| 25 | tr\|Q6S4R7\|Q6S4R7_MEDSA | ADP-ribosylation factor |
| 26 | tr\|O48906\|O48906_MEDSA | malate dehydrogenase |
| 27 | tr\|Q43557\|Q43557_MEDSA | Unidentified |
| 28 | tr\|A0A109RTM3\|A0A109RTM3_MEDSA | Photosystem II CP47 reaction center protein |
| 29 | tr\|A0A4P8F0F5\|A0A4P8F0F5_MEDSA | ATP synthase CF0 B subunit |
| 30 | tr\|Q84U89\|Q84U89_MEDSA | 60S ribosomal protein (Fragment) |
| 31 | tr\|G4W9I5\|G4W9I5_MEDTR | glutamate-1-semialdehyde 2,1-aminomutase |
| 32 | tr\|Q40363\|Q40363_MEDSA | NuM1 protein |
| 33 | tr\|O81692\|O81692_MEDSA | Glycolate oxidase (Fragment) |
| 34 | sp\|Q945F4\|IF5A2_MEDSA | Eukaryotic translation initiation factor 5A-2 |
| 35 | sp\|P46287\|RL11_MEDSA | 60S ribosomal protein L11 |
| 36 | tr\|A0A0X8GKR2\|A0A0X8GKR2_MEDSA | 30S ribosomal protein S4 |
| 37 | tr\|B1P0S0\|B1P0S0_MEDSF | Chlorophyll a-b binding protein, chloroplastic |
| 38 | tr\|A5A4G4\|A5A4G4_MEDSA | Glyceraldehyde-3-phosphate dehydrogenase (Fragment) |
| 39 | tr\|Q45NI6\|Q45NI6_MEDSA | Ribosomal protein L32 |
| 40 | tr\|A0A978W6X0\|A0A978W6X0_MEDSA | Salt tolerant protein |
| 41 | tr\|Q45NH5\|Q45NH5_MEDSA | sucrose synthase (Fragment) |
| 42 | tr\|H6WPX9\|H6WPX9_9BROM | Capsid protein |
| 43 | tr\|B0LSX0\|B0LSX0_MEDSV | Delta-1-pyrroline-5-carboxylate synthase |
| 44 | tr\|A0A0X8GKP6\|A0A0X8GKP6_MEDSA | 30S ribosomal protein S3, chloroplastic |
| 45 | tr\|A0A6B7JET0\|A0A6B7JET0_MEDSF | NAC domain-containing protein NAC46 |
| 46 | tr\|A0A109RT53\|A0A109RT53_MEDSA | Photosystem II CP43 reaction center protein |
| 47 | tr\|A0A109RTT8\|A0A109RTT8_MEDSA | Ribosomal protein L2 |
| 48 | tr\|A0A109RTY1\|A0A109RTY1_MEDSA | Ribosomal protein S7 |
| 49 | tr\|A0A1B1LVP9\|A0A1B1LVP9_MEDSA | Ribosomal protein L20 |
| 50 | tr\|K4MQE6\|K4MQE6_MEDSA | ATP-dependent RNA helicase |
| 51 | tr\|C3VVN9\|C3VVN9_MEDSA | Fructose-bisphosphate aldolase |
| 52 | tr\|A0A109RTN9\|A0A109RTN9_MEDSA | Cytochrome b559 subunit alpha |
| 53 | tr\|Q40368\|Q40368_MEDSA | Rab protein |
| 54 | sp\|P37114\|TCMO_MEDSA | Trans-cinnamate 4-monooxygenase |
| 55 | sp\|P29828\|PDI_MEDSA | Protein disulfide-isomerase |
| 56 | sp\|Q40345\|IDHP_MEDSA | Isocitrate dehydrogenase [NADP], chloroplastic (Fragment) |
| 57 | sp\|P04998\|PSBA_MEDSA | Photosystem II protein D1 |
| 58 | tr\|P93325\|P93325_MEDSA | Delta-1-pyrroline-5-carboxylate synthase (Fragment) |
| 59 | tr\|Q45NN1\|Q45NN1_MEDSA | Pentameric polyubiquitin (Fragment) |
| 60 | tr\|Q9ZT04\|Q9ZT04_MEDSA | 50S ribosomal protein L31 (Fragment) |
| 61 | tr\|B5TXE7\|B5TXE7_9HYPH | ATP synthase beta subunit (Fragment) |
| **62** | **tr\|O24077\|O24077_MEDSA** | **Mitogen-activated protein kinase** |
| **63** | **tr\|Q9FVD4\|Q9FVD4_MEDSV** | **Serine/threonine-protein phosphatase 2A 55 kDa regulatory subunit B** |
| 64 | sp\|Q9XQ94\|GLNA2_MEDSA | Glutamine synthetase leaf isozyme, chloroplastic |
| 65 | tr\|Q45NN8\|Q45NN8_MEDSA | Hydroxyacylglutathione hydrolase (Fragment) |
| 66 | tr\|A9YYK4\|A9YYK4_MEDSA | glucan endo-1,3-beta-D-glucosidase |
| 67 | tr\|W8G2X4\|W8G2X4_MEDSA | GDP-mannose 3,5-epimerase |
| 68 | tr\|A0A120IGV6\|A0A120IGV6_MEDSA | 50S ribosomal protein L16 |
| 69 | tr\|A0A0X8GKM0\|A0A0X8GKM0_MEDSA | Ribosomal protein S19 |
| 70 | tr\|Q40290\|Q40290_MEDSA | CAS15 |
| 71 | tr\|A0A939P6M0\|A0A939P6M0_9HYPH | UDP-N-acetylmuramate--L-alanine ligase |
| 72 | tr\|A0A2V0A0B8\|A0A2V0A0B8_9VIRU | ORF1+2p |
| 73 | tr\|A0A939P3N3\|A0A939P3N3_9HYPH | RNA polymerase sigma factor RpoD |
| **74** | **sp\|Q40353\|MMK2_MEDSA** | **Mitogen-activated protein kinase homolog MMK2** |
| 75 | tr\|A0A1Z1VN40\|A0A1Z1VN40_ENTAG | DNA gyrase subunit B (Fragment) |
| 76 | tr\|A0A0X8GKN1\|A0A0X8GKN1_MEDSA | 30S ribosomal protein S2 |
| 77 | tr\|Q9SP10\|Q9SP10_MEDSA | Glycine-rich RNA binding protein (Fragment) |
| 78 | tr\|Q42921\|Q42921_MEDSA | Phospho-2-dehydro-3-deoxyheptonate aldolase (Fragment) |
| 79 | tr\|Q45NG9\|Q45NG9_MEDSA | Beta-mannosidase |
| 80 | sp\|P50246\|SAHH_MEDSA | Adenosylhomocysteinase |
| 81 | sp\|Q42919\|G6PD_MEDSA | Glucose-6-phosphate 1-dehydrogenase, cytoplasmic isoform |
| 82 | tr\|A0A939P057\|A0A939P057_9HYPH | DNA-directed RNA polymerase subunit beta |
| 83 | tr\|A0A068LJR7\|A0A068LJR7_MEDSA | SIK1 |
| 84 | tr\|A0A939NYR5\|A0A939NYR5_9HYPH | Elongation factor Tu |
| 85 | tr\|A0A939NYN7\|A0A939NYN7_9HYPH | Elongation factor G |
| 86 | tr\|Q9STA4\|Q9STA4_MEDSA | Cysteine protease (Fragment) |
| 87 | tr\|A0A939NZ03\|A0A939NZ03_9HYPH | DeoR/GlpR transcriptional regulator |
| 88 | tr\|B3LE16\|B3LE16_9HYPH | Protein RecA (Fragment) |
| 89 | tr\|A0A193DUD1\|A0A193DUD1_MEDSF | GAMYB-binding protein |
| **90** | **tr\|Q93WR8\|Q93WR8_MEDSV** | **MEK map kinase kinsae** |
| 91 | sp\|P52575\|IFR_MEDSA | Isoflavone reductase |
| 92 | tr\|A0A939NT13\|A0A939NT13_9HYPH | 1,4-alpha-glucan branching enzyme GlgB |
| 93 | tr\|A0A939NVD5\|A0A939NVD5_9HYPH | Amino acid adenylation domain-containing protein |
| 94 | tr\|A0A7J5CMK0\|A0A7J5CMK0_9ACTN | Argininosuccinate synthase |
| 95 | tr\|A0A939P1K8\|A0A939P1K8_9HYPH | Transcription termination factor Rho |
| 96 | tr\|A0A7J5CWI8\|A0A7J5CWI8_9ACTN | Transport permease protein |
| 97 | tr\|A0A1B3T031\|A0A1B3T031_MEDSA | ATP synthase CF1 epsilon subunit |
| 98 | sp\|O82515\|MTDH_MEDSA | Probable mannitol dehydrogenase |
| 99 | tr\|Q8LK12\|Q8LK12_MEDSA | Glyoxysomal malate dehydrogenase (Fragment) |
| 100 | tr\|A0A1B3T011\|A0A1B3T011_MEDSA | Acetyl-coenzyme A carboxylase carboxyl transferase subunit beta, chloroplastic |
| 101 | tr\|A0A0Y0UYF5\|A0A0Y0UYF5_MEDSA | NADH-quinone oxidoreductase subunit D |
| 102 | tr\|A0A939P3A4\|A0A939P3A4_9HYPH | 50S ribosomal subunit assembly factor BipA |
| 103 | tr\|Q40323\|Q40323_MEDSA | 70 kD heatshockprotein (Fragment) |
| **104** | **tr\|Q9FVD6\|Q9FVD6_MEDSV** | **Ser/Thr specific protein phosphatase 2A A regulatory subunit beta isoform** |
| 105 | tr\|Q2VRR7\|Q2VRR7_MEDSA | Ribosomal protein S12 (Fragment) |
| 106 | tr\|Q9XF99\|Q9XF99_MEDSA | Skp1 (Fragment) |
| 107 | tr\|Q9SXZ9\|Q9SXZ9_MEDSA | Uricase |
| 108 | sp\|P51139\|MSK3_MEDSA | Glycogen synthase kinase-3 homolog MsK-3 |
| 109 | tr\|Q45NH1\|Q45NH1_MEDSA | DNA-directed RNA polymerase II third largest subunit (Fragment) |
| 110 | sp\|P49163\|RK22_MEDSA | 50S ribosomal protein L22, chloroplastic |
| 111 | sp\|P28014\|TCTP_MEDSA | Translationally-controlled tumor protein homolog |
| 112 | tr\|Q93XK5\|Q93XK5_MEDSA | Peroxidase |
| 113 | tr\|A0A6H9X1H6\|A0A6H9X1H6_9ACTN | 50S ribosomal protein L28 |
| **114** | **sp\|Q07176\|MMK1_MEDSA** | **Mitogen-activated protein kinase homolog MMK1** |
| 115 | tr\|A0A7J5CWY1\|A0A7J5CWY1_9ACTN | UDP-glucuronate decarboxylase |
| 116 | tr\|Q66PN2\|Q66PN2_MEDSA | Sucrose-phosphatase (Fragment) |
| 117 | tr\|A0A7J5ATT4\|A0A7J5ATT4_9ACTN | Adenylate/guanylate cyclase domain-containing protein |
| 118 | tr\|A0A939SZC9\|A0A939SZC9_9HYPH | Isovaleryl-CoA dehydrogenase |
| 119 | tr\|A8DX61\|A8DX61_MEDSA | DH OS=Medicago sativa |
| 120 | tr\|A0A939P041\|A0A939P041_9HYPH | 30S ribosomal protein S3 |
| 121 | tr\|G9JZL3\|G9JZL3_MEDSA | Putative nuclear acid binding protein |
| 122 | tr\|P94084\|P94084_MEDSA | chitinase |
| 123 | tr\|A0A109RU04\|A0A109RU04_MEDSA | DNA-directed RNA polymerase subunit alpha |
| 124 | tr\|A0A0X8GKJ8\|A0A0X8GKJ8_MEDSA | NADH dehydrogenase subunit 7 |
| 125 | tr\|A0A120IGV7\|A0A120IGV7_MEDSA | Ribosomal protein L14 |
| 126 | tr\|A0A939P0P1\|A0A939P0P1_9HYPH | ATP-dependent protease ATPase subunit HslU |
| 127 | tr\|G4XKY1\|G4XKY1_9CARY | V-type proton ATPase proteolipid subunit |
| 128 | tr\|Q9M603\|Q9M603_MEDSA | Cold acclimation responsive protein BudCAR5 |
| 129 | tr\|Q45NL6\|Q45NL6_MEDSA | sucrose synthase (Fragment) |
| 130 | tr\|A4ZUE4\|A4ZUE4_MEDSF | Sucrose synthase |
| 131 | tr\|A0A6H9XIW5\|A0A6H9XIW5_9ACTN | 30S ribosomal protein S12 |
| 132 | sp\|O65026\|SUS_MEDSA | Sucrose synthase |
| 133 | tr\|A0A7J5CZ20\|A0A7J5CZ20_9ACTN | Elongation factor G |
| 134 | tr\|A0A8F2Z101\|A0A8F2Z101_9VIRU | Coat protein |
| 135 | tr\|A0A7J5ATX6\|A0A7J5ATX6_9ACTN | DNA-directed RNA polymerase subunit beta' |
| 136 | tr\|Q6DW08\|Q6DW08_MEDSA | mannose-1-phosphate guanylyltransferase |
| 137 | tr\|A0A7J5CYM7\|A0A7J5CYM7_9ACTN | Polyribonucleotide nucleotidyltransferase |
| 138 | tr\|A0A939T142\|A0A939T142_9HYPH | Ribose-phosphate pyrophosphokinase |
| 139 | tr\|H2EIY6\|H2EIY6_MEDSA | Spermidine synthase |
| 140 | tr\|A0A109RSY0\|A0A109RSY0_MEDSA | Photosystem II D2 protein |
| 141 | sp\|Q9ZT05\|PSAK_MEDSA | Photosystem I reaction center subunit psaK, chloroplastic |
| 142 | tr\|Q40311\|Q40311_MEDSA | 6-phosphogluconate dehydrogenase, decarboxylating |
| 143 | tr\|A0A939P2U0\|A0A939P2U0_9HYPH | Fructose-bisphosphate aldolase |
| 144 | tr\|A0A2Z4KH80\|A0A2Z4KH80_MEDSA | Ribosomal protein S14 |
| 145 | tr\|A0A6H9X5C0\|A0A6H9X5C0_9ACTN | ATP-dependent Clp protease ATP-binding subunit |
| 146 | tr\|A0A120IH28\|A0A120IH28_MEDSA | Ribosomal protein S8 |
| 147 | tr\|A0A7J4ZXX2\|A0A7J4ZXX2_9ACTN | DEAD/DEAH box helicase |
| 148 | tr\|A0A385NFP2\|A0A385NFP2_MEDSA | assimilatory sulfite reductase (ferredoxin) |
| 149 | tr\|A0A7J5CNI4\|A0A7J5CNI4_9ACTN | Ricin B lectin domain-containing protein |
| 150 | tr\|A0A939P105\|A0A939P105_9HYPH | Sn-glycerol-3-phosphate ABC transporter ATP-binding protein UgpC |
| 151 | tr\|A0A7J5CZ46\|A0A7J5CZ46_9ACTN | Elongation factor Tu |
| 152 | tr\|A0A6H9X9J0\|A0A6H9X9J0_9ACTN | GTPase Era |
| 153 | tr\|A0A7J5CSG0\|A0A7J5CSG0_9ACTN | Chaperone protein HtpG |
| 154 | tr\|A0A0X8GKL0\|A0A0X8GKL0_MEDSA | 30S ribosomal protein S18, chloroplastic |
| 155 | tr\|A0A0Y0URF0\|A0A0Y0URF0_MEDSA | H(+)-transporting two-sector ATPase |
| 156 | tr\|Q9S967\|Q9S967_MEDSA | Calcium-dependent protein kinase 2 (Fragment) |
| 157 | tr\|A0A939T2J0\|A0A939T2J0_9HYPH | Bifunctional protein PutA |
| 158 | tr\|A0A939NY42\|A0A939NY42_9HYPH | ATP-dependent Clp protease ATP-binding subunit ClpA |
| 159 | sp\|P04078\|GLNA1_MEDSA | Glutamine synthetase cytosolic isozyme |
| 160 | tr\|A0A978W6Z3\|A0A978W6Z3_MEDSA | Drought tolerant protein |
| 161 | tr\|A0A939NX28\|A0A939NX28_9HYPH | Biotin-dependent carboxyltransferase family protein |
| 162 | tr\|G8D4Z4\|G8D4Z4_MEDSA | Ribulose bisphosphate carboxylase large chain (Fragment) |
| 163 | sp\|Q05006\|CDC22_MEDSA | Cell division control protein 2 homolog 2 |
| 164 | tr\|A0A0X8GJX4\|A0A0X8GJX4_MEDSA | 50S ribosomal protein L20, chloroplastic |
| 165 | tr\|A8IFN5\|A8IFN5_MEDSA | Mitochondrial S1 ribosomal protein |

**Table S3 Prediction of potential phosphorylation sites in MsNAC73 by GPS (http://gps.biocuckoo.cn).**

| ID | Position | Code | Kinase | PSP | Score | Cutoff |
| --- | --- | --- | --- | --- | --- | --- |
| MsNAC73protein | 22 | S | AGC | LMERRKDSLIRTCPT | 0.0689 | 0.0147 |
| MsNAC73protein | 253 | T | AGC | GFVEYYSTSFISFDQ | 0.0206 | 0.0147 |
| MsNAC73protein | 29 | T | CK1 | SLIRTCPTCGHHIKC | 0.0937 | 0.0368 |
| MsNAC73protein | 97 | T | CK1 | GENGICCTHPEKLPG | 0.0478 | 0.0368 |
| MsNAC73protein | 119 | S | CK1 | RHFFHRPSKAYTTGT | 0.0408 | 0.0368 |
| MsNAC73protein | 124 | T | CK1 | RPSKAYTTGTRKRRK | 0.133 | 0.0368 |
| MsNAC73protein | 126 | T | CK1 | SKAYTTGTRKRRKVH | 0.0602 | 0.0368 |
| MsNAC73protein | 139 | S | CK1 | VHTDADGSETRWHKT | 0.0985 | 0.0368 |
| MsNAC73protein | 141 | T | CK1 | TDADGSETRWHKTGK | 0.0513 | 0.0368 |
| MsNAC73protein | 146 | T | CK1 | SETRWHKTGKTRPVF | 0.0532 | 0.0368 |
| MsNAC73protein | 209 | T | CK1 | VSKVFYQTQPRQCGG | 0.1139 | 0.0368 |
| MsNAC73protein | 222 | S | CK1 | GGSLMKDSASFSDKK | 0.209 | 0.0368 |
| MsNAC73protein | 245 | S | CK1 | EVVNHKNSGFVEYYS | 0.0592 | 0.0368 |
| MsNAC73protein | 252 | S | CK1 | SGFVEYYSTSFISFD | 0.0742 | 0.0368 |
| MsNAC73protein | 254 | S | CK1 | FVEYYSTSFISFDQG | 0.107 | 0.0368 |
| MsNAC73protein | 266 | S | CK1 | DQGEQHRSNNAQVIS | 0.0575 | 0.0368 |
| MsNAC73protein | 29 | T | CMGC | SLIRTCPTCGHHIKC | 0.0179 | 0.0149 |
| MsNAC73protein | 97 | T | CMGC | GENGICCTHPEKLPG | 0.0165 | 0.0149 |
| MsNAC73protein | 119 | S | CMGC | RHFFHRPSKAYTTGT | 0.0416 | 0.0149 |
| MsNAC73protein | 124 | T | CMGC | RPSKAYTTGTRKRRK | 0.0463 | 0.0149 |
| MsNAC73protein | 126 | T | CMGC | SKAYTTGTRKRRKVH | 0.0333 | 0.0149 |
| MsNAC73protein | 149 | T | CMGC | RWHKTGKTRPVFVSG | 0.0164 | 0.0149 |
| MsNAC73protein | 155 | S | CMGC | KTRPVFVSGKLKGYK | 0.0422 | 0.0149 |
| MsNAC73protein | 217 | S | CMGC | QPRQCGGSLMKDSAS | 0.02 | 0.0149 |
| MsNAC73protein | 245 | S | CMGC | EVVNHKNSGFVEYYS | 0.0187 | 0.0149 |
| MsNAC73protein | 254 | S | CMGC | FVEYYSTSFISFDQG | 0.0272 | 0.0149 |
| MsNAC73protein | 22 | S | STE | LMERRKDSLIRTCPT | 0.9224 | 0.6198 |
| MsNAC73protein | 123 | T | TKL | HRPSKAYTTGTRKRR | 0.4498 | 0.4268 |
| MsNAC73protein | 224 | S | TKL | SLMKDSASFSDKKLN | 0.4535 | 0.4268 |
| MsNAC73protein | 6 | Y | TK | **MTQCSYPENNHST | 0.888 | 0.5005 |
| MsNAC73protein | 122 | Y | TK | FHRPSKAYTTGTRKR | 0.9708 | 0.5005 |
| MsNAC73protein | 187 | Y | TK | TNWVMHQYHLGNDEE | 0.864 | 0.5005 |
| MsNAC73protein | 207 | Y | TK | LVVSKVFYQTQPRQC | 0.7427 | 0.5005 |
| MsNAC73protein | 250 | Y | TK | KNSGFVEYYSTSFIS | 0.5651 | 0.5005 |
| MsNAC73protein | 251 | Y | TK | NSGFVEYYSTSFISF | 0.7602 | 0.5005 |

**Table S4 Potential phosphorylation sites of MsNAC73 analyzed by LC-MS/MS.**

| Position | Enzyme | Peptide < Protein Metrics Confidential > | Modification Type(s) | Observed m/z | z | Observed (M+H) | Calc. mass (M+H) | Mass error (ppm) | Starting position | Score | Scan Time | Intensity |
| --- | --- | --- | --- | --- | --- | --- | --- | --- | --- | --- | --- | --- |
| 123 | TG | R.PSKAY**T**[+79.966]TGTR.K | T[+80] | 581.279 | 2 | 1161.550 | 1161.530 | 17.5 | 356 | 35.3 | 26.5303 | 243120000 |
| 245 | TG | E.VVNHKN**S**[+79.966]GFVE.Y | S[+80] | 655.311 | 2 | 1309.614 | 1309.594 | 15.8 | 477 | 30.0 | 31.7007 | 59071000 |
| 22 | TG | R.RKD**S**[+79.966]LIR.T | S[+80] | 484.253 | 2 | 967.498 | 967.509 | -10.8 | 257 | 10.8 | 6.4128 | 12383000 |

**Table S5 Primers used in this study.**

| ﻿Primer name | ﻿Sequence 5’ to 3’ | Purpose |
| --- | --- | --- |
| ﻿MsPG2-F | ATGCAGGACTTGTTTATATCTG | ﻿﻿Gene clone |
| ﻿MsPG2-R | TTACTGGTGTAGACAGTGGAAGGGA |  |
| ﻿MsPG2-YFP-F | GCTTGGATCCTCGAGCTGCAGATGCAGGACTTGTTTATATCTG | Plant transformation and ﻿Subcellular localization |
| ﻿MsPG2-YFP-R | GCCCTTGCTCACCATACTAGTCTGGTGTAGACAGTGGAAG |  |
| MsPG2-Flag-F | CTTGATATCGAATTCCTGCAGATGCAGGACTTGTTTATATCTG |  |
| MsPG2-Flag-R | TTATCGATACCGTCGGATCCCTGGTGTAGACAGTGGAAGGGA |  |
| MsPG2-Ri-F | ACAATATCATGGTCAATCAA | Plant transformation |
| MsPG2-Ri-R | CTGGTGTAGACAGTGGAAGGGA |  |
| MsPG2-pTOPO-F | GAAGGAGCCCTTCACCGGATCCACAATATCATGGTCAATCAA | Plant transformation |
| MsPG2-pTOPO-R | CGGGTTTGAGCTCAAAGAATTCCTGGTGTAGACAGTGGAAGGGA |  |
| ﻿MsPG2q-F | ATGGTCAGGGTGAAGCTTGG | ﻿RT-qPCR |
| ﻿MsPG2q-R | AGCAATGCAATCATCGCCAC |  |
| ﻿MsEF-α-F | ﻿GCACCAGTGCTCGATTGC |  |
| ﻿MsEF-α-R | ﻿TCGCCTGTCAATCTTGGTAACAA |  |
| MsActin2q-F | CCATTGAGCACGGTATTGTC |  |
| MsActin2q-R | ATTGGCCTTTGGGTTAAGTG |  |
| MsPG2pro-F | AACTCTTGGGAGGGCAATGT | ﻿﻿Gene clone |
| MsPG2pro-R | TTGGCACCATATTGAAGCACG |  |
| MsPG2pro-pLacZ-F1 | TGATATTGGATCGGAATTCAACTCTTGGGAGGGCAAT | ﻿Y1H assay |
| MsPG2pro-pLacZ-R | ACAGAGCACATGCCTCGAGGATGAATATATTATGTTA |  |
| MsPG2pro-pLacZ-F2 | TGATATTGGATCGGAATTCTTAGAGGAAAAACCTT |  |
| MsPG2pro-pLacZ-F3 | TGATATTGGATCGGAATTCTCTCTTGCATCATAAGTA |  |
| MsPG2pro-pLacZ-F4 | TGATATTGGATCGGAATTCCCTCCGGTTGAAATATGAATC |  |
| MsPG2pro-pLacZ-F5 | TGATATTGGATCGGAATTCGCAAGGTTGCACATATTAAACC |  |
| MsPG2pro-pLacZ-F6 | TGATATTGGATCGGAATTCCCATGTCTATATACTATAAG |  |
| MsPG2pro-pLacZ-F7 | TGATATTGGATCGGAATTCCATGGTTAGTGAACATATAC |  |
| MsNAC73-pB42AD-F | TGCCTCTCCCGAATTCATGACTCAGTGTAGTTACCC |  |
| MsNAC73-pB42AD-R | TCCAAAGCTTCTCGAGTCAAGGAATGAAAGAAGTAC |  |
| MsPG2pro(3×TC)-pLacZ-F | AATTCACACTTgttagaaaatACATATACACTTgttagaaaatACATATACACTTgttagaaaatACATATC |  |
| MsPG2pro(3×TC)-pLacZ-R | TCGAGATATGTattttctaacAAGTGTATATGTattttctaacAAGTGTATATGTattttctaacAAGTGTG |  |
| MsPG2pro(3×mTC)-pLacZ-F | AATTCACACTTACATATACACTTACATATACACTTACATATC |  |
| MsPG2pro(3×mTC)-pLacZ-R | TCGAGATATGTAAGTGTATATGTAAGTGTATATGTAAGTGTG |  |
| MsNAC73-pCold-F | ﻿CGAAGGTAGGCATATGATGACTCAGTGTAGTTACCC | EMSA assay and pull-down assay |
| MsNAC73-pCold-R | ﻿AGATTACCTATCTAGATCAAGGAATGAAAGAAGTAC |  |
| MsPG2pro-﻿pGreen0800-F | TTGATATCGAATTCCTGCAGTCTCTTGCATCATAAGTA | ﻿Dual-LUC assay |
| MsPG2pro-﻿pGreen0800-R | GCTCTAGAACTAGTGGATCCGATGAATATATTATGTTA |  |
| MsNAC73-Flag-F | CTTGATATCGAATTCCTGCAGATGACTCAGTGTAGTTACCC | ﻿Dual-LUC assay and Co-IP |
| MsNAC73-Flag-R | TTATCGATACCGTCGGATCCAGGAATGAAAGAAGTAC |  |
| MsNAC73-Ri-F | TACCATCTTGGAAATGATGA | Plant transformation |
| MsNAC73-Ri-F | AGGAATGAAAGAAGTACCATC |  |
| MsNAC73-pTOPO-F | GAAGGAGCCCTTCACCGGATCCTACCATCTTGGAAATG | Plant transformation |
| MsNAC73-pTOPO-R | CGGGTTTGAGCTCAAAGAATTCAGGAATGAAAGAAGTAC |  |
| ﻿MsNAC73q-F | TCTACCAAACACAGCCTAGACA | ﻿RT-qPCR |
| ﻿MsNAC73q-R | AACCACCTCATGCACACCTTG |  |
| MsMPK3-F | ATGGCCGGAGTTAACCAA | ﻿﻿Gene clone |
| MsMPK3-R | TTAAGCATACTCAGGATTGAGTGC |  |
| ﻿MsMPK3-YFP-F | GCTTGGATCCTCGAGCTGCAGATGGCCGGAGTTAACCAA | Dual-LUC assay, Co-IP and ﻿Subcellular localization |
| ﻿MsMPK3-YFP-R | GCCCTTGCTCACCATACTAGTAGCATACTCAGGATTGAG |  |
| MsNAC73-cLUC-F | GTCCCGGGGCGGTACCATGACTCAGTGTAGTTAC | Split-LUC assay |
| MsNAC73-cLUC-R | AGCTCTGCAGGTCGACTCAAGGAATGAAAGAAGT |  |
| MsMPK3-nLUC-F | GGACGAGCTCGGTACCATGGCCGGAGTTAACCAA |  |
| MsMPK3-nLUC-R | ACGAGATCTGGTCGACAGCATACTCAGGATTGAG |  |
| MsNAC73-nYFP-F | ATCGAGGACGCCGGCGGATCCATGACTCAGTGTAGTTACC | BiFC assay |
| MsNAC73-nYFP-R | ACGAAAGCTCTGCAGGTCGAC TCAAGGAATGAAAGAAGTA |  |
| MsMPK3-cYFP-F | TTACAATTACAGGTACCCGGG ATGGCCGGAGTTAACCAA |  |
| MsMPK3-cYFP-R | GCCACCGCCGTCGACTCTAGA AGCATACTCAGGATTGAG |  |
| ﻿MsMPK3q-F | GTTTCCGGCGGTTCAAACTC | ﻿RT-qPCR |
| ﻿MsMPK3q-R | CAATCGGCATGATCGGAGGA |  |
| MsNAC73-a | ATGACTCAGTGTAGTTACCC | Overlap-PCR Phosphomimetic ﻿form and Phosphorylation-deficient ﻿form |
| MsNAC73-d | TCAAGGAATGAAAGAAGTAC |  |
| MsNAC73^T123A^-b | AAGCATACGCCACAGGAACA |  |
| MsNAC73^T123A^-c | TGTTCCTGTGGCGTATGCTT |  |
| MsNAC73^T123D^-b | AAGCATACGACACAGGAACA |  |
| MsNAC73^T123D^-c | TGTTCCTGTGTCGTATGCTT |  |
